# Analysis of Scientific and Press Articles Related to Cultured Meat for a Better Understanding of Its Perception

**DOI:** 10.3389/fpsyg.2020.01845

**Published:** 2020-08-25

**Authors:** Sghaier Chriki, Marie-Pierre Ellies-Oury, Dominique Fournier, Jingjing Liu, Jean-François Hocquette

**Affiliations:** ^1^ISARA, Agroecology and Environment Unit, Lyon, France; ^2^Bordeaux Sciences Agro, Gradignan, France; ^3^INRAE, Clermont-Ferrand, VetAgro Sup, Saint Genès Champanelle, France; ^4^INRAE, SDAR, Montpellier, France

**Keywords:** cultured meat, Web of Science, press, public, perception, bibliometrics

## Abstract

Cultured meat is presented by its advocates as a good alternative for consumers who want to be more ethically minded but who do not wish to change their diet. This novel food has become an emerging topic in both the scientific field and the press media. From a bibliometric analysis of scientific publications and on a sociometric analysis of the mainstream press, the aim of this study was to identify potential differences between the scientific view and the public perception. This research analyzed the publications indexed by SCI-EXPANDED in the Web of Science Core Collection database owned by Clarivate Analytics, for scientific literature analysis, and indexed by the Factiva database, for the press media. A total of 327 scientific publications were analyzed according to year of publication and country and institution of origin, also including coauthorships, co-citations, and scientific fields' and journals' networks. A knowledge mapping using VOSviewer was used to study the literature in the field. Based on Factiva, 12,900 press articles dealing with artificial meat, mainly in English, have been found through public databases. The main conclusion is that cultured meat is mainly developing in the USA and the UK, with other countries, such as China, observing the trend for potential future applications. Scientific articles seemed initially to focus mainly on technical aspects of artificial meat and more recently on health value, consumer's acceptance, and sustainability. However, the potential environment-friendly effects of this novel food are more and more studied or described in scientific or press articles.

## Introduction

Besides animal farming, many efficient ways of protein production are being developed to satisfy the increasing demand for food by the growing human population, while taking into account today's challenges when it comes to livestock, may they be environmental or in terms of animal welfare (Scollan et al., 2011; Aiking, 2014; Gerber et al., 2015; Willett et al., 2019). Among the solutions, cultured meat or *in vitro* meat is particularly promoted by its advocates as a sustainable alternative for consumers who want to be more ethically minded but who do not wish to change the composition of their diet (Post, 2012; Kadim et al., 2015; Moritz et al., 2015; Shapiro, 2018; Chriki and Hocquette, 2020).

Pros and cons of the cultured meat process were recently described in a review Chriki and Hocquette (2020). In this review, the authors updated current knowledge on this subject by focusing on recent publications and issues, which had not been well-described previously.

In August 2013, the first “lab-grown hamburger” was prepared and tasted during a television program (Post, 2014). Since then, the rise of the global cultured meat market has been heralded. Consequently, this novel food has attracted a lot of media attention, but the treatment has been vastly different depending on the media. Particularly, some scientists (Goodwin and Shoulders, 2013; Hopkins, 2015) concluded that the Western media have given a distorted picture of the obstacles which are in the path of cultured meat acceptance, especially by overemphasizing and over representing the importance of the reception of cultured meat among vegetarians.

In this context, the aim of this study was to understand how the topic of cultured meat is treated in the scientific literature and in the news media to identify potential differences between the scientific view and the public perception. Thus, this study was based on a bibliometric analysis of scientific publications and on a sociometric analysis of the mainstream press about *in vitro* meat.

## Methodology

Using academic databases to conduct research on specialized topics has become the normative mode of scholarly investigation (Fernandes et al., 2019). Electronic databases that gather scientific publications provide a mechanism for rapid access to broad information, eliminating the need to manually search through paper copies of various publication types (Driedger and Weimer, 2015).

Characterized as a functional way to measure the influence of publications in scientific communities, **bibliometric analysis** is defined as “a statistical analysis of books, scientific articles, or other media of communication” (Pritchard, 1969, p. 349). Indeed, the academic impact of any research (or of a specific article) can be assessed by the number of citations by other authors in the specific field (Iftikhar et al., 2019). However, other analyses can be conducted using the available research filters by year or country of publication or using keywords for example (Fernandes et al., 2019). For articles from the written press, similar analyses can be conducted as well (Goodwin and Shoulders, 2013; Hopkins, 2015).

### Data Sources

This study on **cultured meat** was based on the science literature from the Science Citation Index Expanded (SCI-EXPANDED) database of the Web of Science (WoS) Core Collection database from Clarivate Analytics (formerly known as the Institute for Scientific Information). Using WoS as the search source provided researchers with quality literature and gave solid basis to the study (Jacso, 2005; Zhao et al., 2019; Zhu and Liu, 2020). Some comparative studies concluded that WoS and Scopus retrieved no duplicates, while Google Scholar retrieved multiple copies (Adriaanse and Rensleigh, 2013; Driedger and Weimer, 2015).

Indeed, WoS covers a wide range of studies and thus offers a more general and comparative view of publications in specific fields (here, cultured meat).

In order to compare citation impact for published papers, data were sent to InCites, which provides normalized citation data and global metrics from the WoS dataset.

The following analysis was performed: coauthorship (the relatedness of items is based on the number of coauthored documents) and co-citation (the relatedness of items is based on the number of times they cite each other) (van Eck and Waltman, 2010).

For the written press, this study was carried out with the Factiva database, produced by the Dow Jones (Johal, 2009; Driedger and Weimer, 2015). This business information and research tool provides worldwide, full-text coverage of international newspapers and newswires which helps researchers to carry out an information watch and analyze media coverage on a specific subject (Chen et al., 2020). The units selected for content analysis using an interface of R (R Core Team, 2018), named IRaMuTeQ, were articles published in daily newspapers from 2010 to 2019 with a title and a full text in English or with at least a title translated into English. Based on R software and python language, IRaMuTeQ extracted qualitative information from texts (such as keywords) using descriptive statistics (Chaves et al., 2017).

Other specific platforms such as the *China National Knowledge Infrastructure* (CNKI) (cnki.net) and the *Baidu Scholar* platform, which are the most widely used platforms in China, were also used to specifically target Chinese publications. Different names designing artificial meat used in English publications were translated into Chinese and used as keywords to extract corresponding articles through titles, keywords, and full texts. The number of press articles was collected according to the publication year and article type. A general understanding of the main perspective of articles dealing with artificial meat was therefore obtained and analyzed as for the English ones.

### Keyword Selection

The 24 keywords used to collect publications (Table 1) were based on scientific articles and reviews dealing with cultured meat, particularly those based on the influence of the name on the acceptance of this novel food (Siegrist and Sütterlin, 2017; Asioli et al., 2018; Siegrist et al., 2018; Bryant and Barnett, 2019; Bryant C. J. et al., 2019; Ong et al., 2020). The question whether these keywords cover most of the articles from the written press will be discussed later based on the results.

**Table 1:** Different names of cultured meat used in scientific publications.

**Names/keywords**	**References^b^**
Cultured meat (97)^a^	Edelman et al., 2005; Bhat and Fayaz, 2011; Forgacs et al., 2012; Post, 2012; Hopkins, 2015; Bryant and Barnett, 2018; Hamdan et al., 2018; Bodiou et al., 2020; Chriki and Hocquette, 2020; Weinrich et al., 2020; Zhang et al., 2020
*in vitro* meat (85)	Datar and Betti, 2010; Laestadius, 2015; Sharma et al., 2015; Hocquette, 2016; Wilks and Phillips, 2017; Lee, 2018; Bhat et al., 2019; Bryant and Barnett, 2019; Woll, 2019; Li et al., 2020
Clean meat (25)	Lagally and Specht, 2017; Windhorst, 2018, 2019; Bryant C. et al., 2019; Bryant C. J. et al., 2019
Artificial meat (21)	Bonny et al., 2015, 2017; Hocquette, 2015; Hocquette et al., 2015; Orzechowski, 2015; Sodhi, 2017
Synthetic meat (19)	Kadim et al., 2015; Marcu et al., 2015; Jones, 2017; Siegrist and Sütterlin, 2017; Lynch and Pierrehumbert, 2019; Warner, 2019
Cell-based meat (10)/cell-cultured meat (1)/cellular meat (1)	Bomgardner, 2018b; Johnson, 2019; Mohorcich and Reese, 2019; Simsa et al., 2019; Swartz, 2019; Warner, 2019
Lab-grown meat (7)/lab meat (2)	Galusky, 2014; Mayhall, 2019; Mouat et al., 2019; Warner, 2019
Fake meat (11)	Fellet, 2015; Grimstead, 2018; Bomgardner, 2019
Vegetarian (8)/vegan meat (3)	Hopkins, 2015; Weber, 2018; Alvaro, 2019
Animal-free meat (5)	Bhat et al., 2017; Bomgardner, 2018a; Mouat et al., 2019
Test tube meat (4)	Fox, 2009
Cultivated meat (3)	Borning and Tiberius, 2017
Other names: craft meat, victimless meat, cruelty-free meat, slaughter-free meat, Frankenmeat, unnatural meat, shmeat	Metcalf, 2013; Welin, 2013; Marcu et al., 2015; Wilks and Phillips, 2017; Siegrist et al., 2018; Alvaro, 2019; Bhat et al., 2019; Bryant and Barnett, 2019; Burton, 2019; Mouat et al., 2019; Ong et al., 2020

a *Names/keywords' number of citations in titles, keywords, and abstracts of articles*.

b *This reference list is not exhaustive: the articles indicated as examples are those mainly discussed in the Results section*.

Both in WoS (in *Topic*, as of December 31, 2019) and Factiva (as of December 31, 2019) databases, we searched for articles containing the following words:

“*artificial meat*” *OR* “*meat in vitro*” *OR* “*in vitro meat*” *OR* “*cultured meat*” *OR* “*synthetic meat*” *OR* “*lab-grown meat*” *OR* “*lab meat*” *OR* “*cell-based meat*” *OR* “*clean meat*” *OR* “*fake meat*” *OR* “*slaughter-free meat*” *OR “cell-cultured meat” OR “craft meat” OR “cultivated meat” OR “victimless meat” OR “animal-free meat” OR “cruelty-free meat” OR “shmeat” OR “Frankenmeat” OR “test tube meat” OR “unnatural meat” OR “vegetarian meat” OR “vegan meat” OR “cellular meat*.”

### Data Analysis

Among others, we considered different sets of elements that characterize the scientific or the press publications, such as year, scientific fields, journal, and authors, etc., to analyze data collected from WoS and/or Factiva.

The obtained results were analyzed by means of univariate statistics (absolute and relative frequency) and compared with what was postulated by the Laws of Bibliometrics, namely, Lotka's Law, Bradford's Law, and Zipf's Law based on authors' production on the studied topic, journal coverage of the topic, or occurrence of keywords related to the subject, respectively (Fernandes et al., 2019; Zhao et al., 2019). This allowed to identify patterns and to trace possible biases for this subject in the academic field or in mainstream media.

### VOS Mapping

Then, for scientific articles (from WoS) only, the production of maps structured through the VOS mapping technique was used, according to Korom (2019). The construction of a VOS map basically follows three steps, developed by the VOSviewer software: normalization, mapping, and clustering.

VOSviewer is a very useful tool for graphical representation of bibliometric maps. This software, available for free, offers a convenient process for constructing and visualizing bibliometric maps of any kind of co-occurrence data (van Eck and Waltman, 2010).

## Results

### Scientific Articles Dealing With Cultured Meat From the Web of Science Database

#### Time Distribution and Scientific Fields' Networks

**Figure 1:**
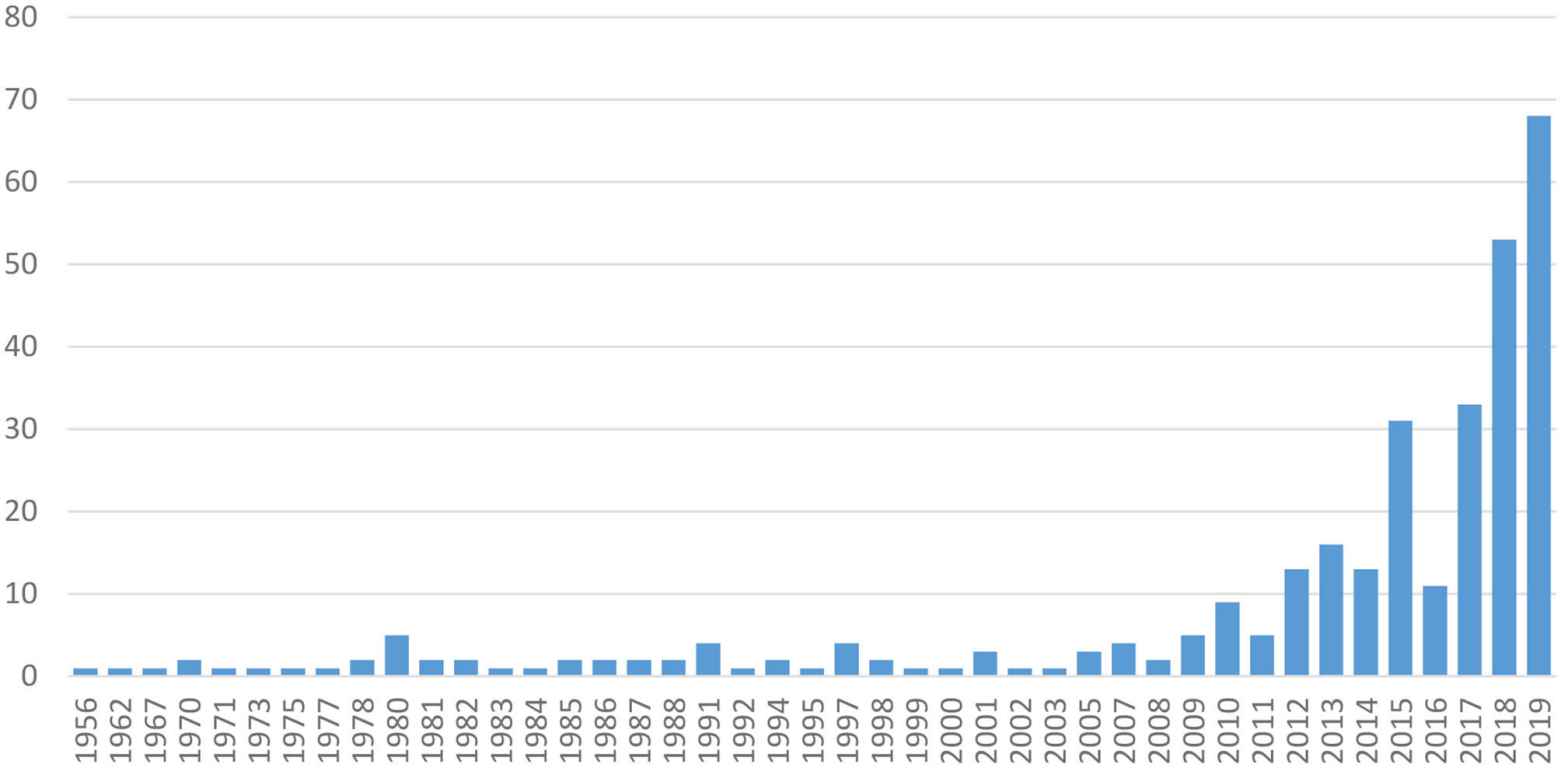
Number of articles dealing with cultured meat recorded per year (as of December 31, 2019) in the Web of Science (WoS) bibliometric database.

A total of 327 publications from the WoS (see Supplmentary Material) were collected and further analyzed. After some papers mentioning words related with synthetic meat, a first significant increase in the number of scientific papers dealing with cultured meat was observed in 2012–2014, then in 2015. From 2017, the number of papers dealing with cultured meat has regularly increased (Figure 1).

**Figure 2:**
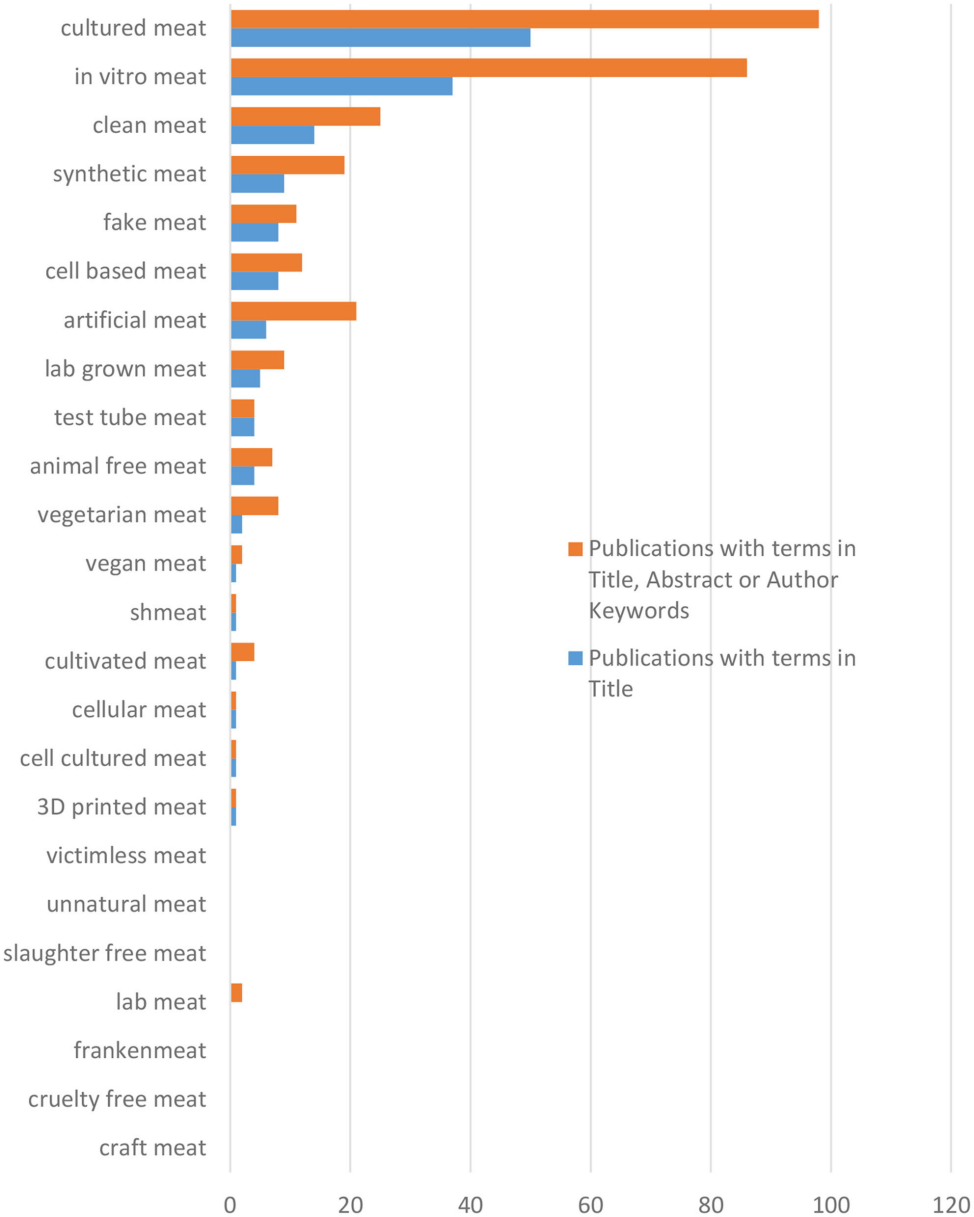
Major keywords used in the scientific literature [in the Web of Science (WoS)] to designate *in vitro* meat.

Within the 24 keywords studied in this bibliometric analysis, two of them were the most widely used, namely, “cultured meat,” and to a lesser extent, “*in vitro* meat” (Figure 2).

Quite logically, the main scientific field in which scientific articles about cultured meat were published is *Food Science Technology* (Table 2). Indeed, these articles mainly concern the process of cultured meat. However, a significant proportion of articles also concerns nutritional or environmental issues, agricultural science or social science, such as history, philosophy of sciences, or ethics (Table 2).

**Table 2:** Major scientific fields in which articles related to cultured meat were published.

**Major web of science categories**	**Number of publications**
Food Science and Technology	86
Nutrition and Dietetics	32
Environmental Sciences	30
Agriculture Multidisciplinary	27
History and Philosophy of Science	26
Applied Microbiology and Biotechnology	25
Agriculture, Dairy and Animal Science	21
Multidisciplinary Sciences	21
Ethics	17
Cell Biology	13
Behavioral Sciences	11
Chemistry Multidisciplinary	11

**Figure 3:**
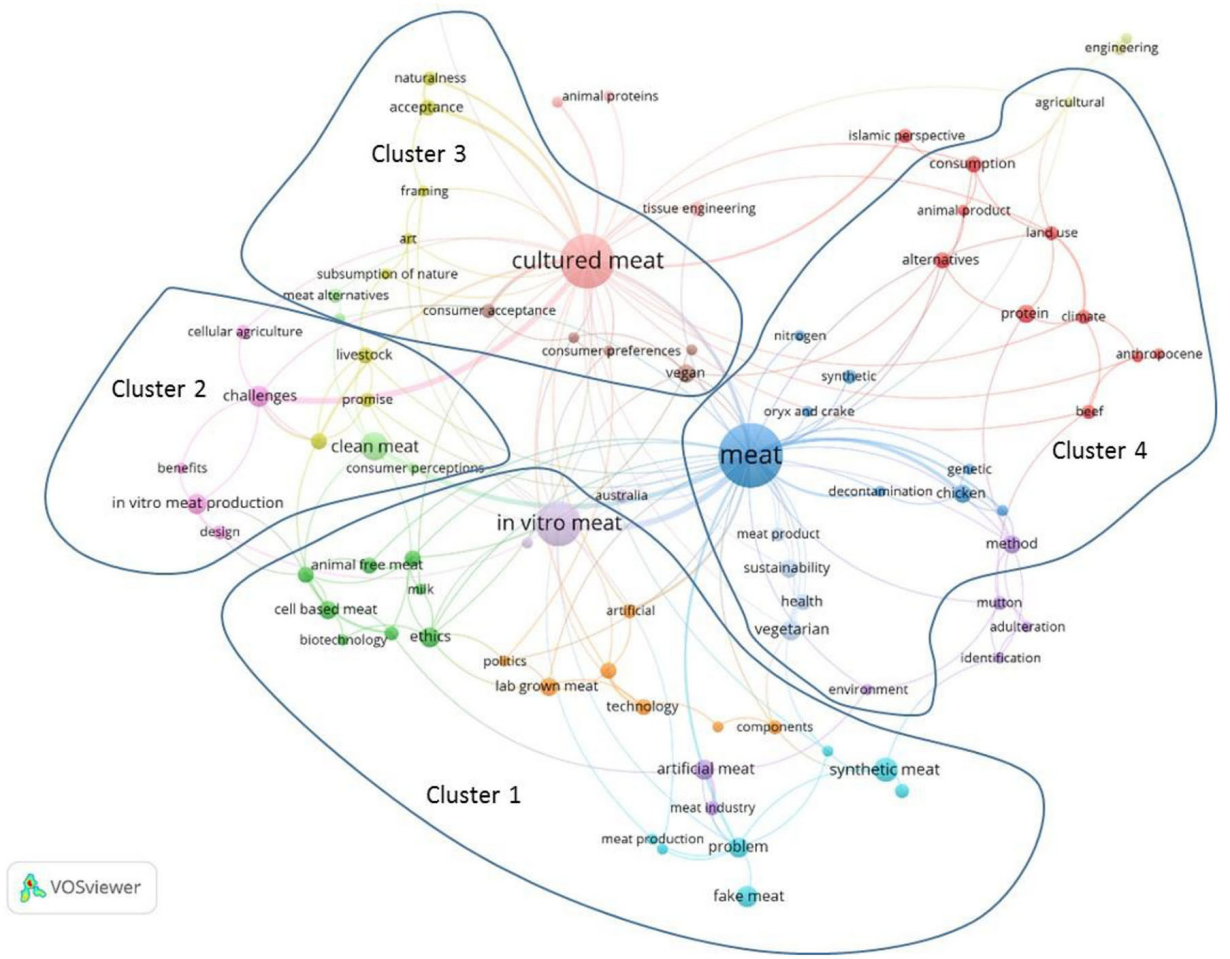
Keywords co-occurrence network of predominant terms in Web of Science (WoS) publications dealing with cultured meat.

This view was confirmed by a more precise analysis of relationships between keywords in titles, author keywords, and abstracts. With the 97 keywords found in the scientific articles, four peripheral networks or clusters surrounding the most common wordings were observed. **Cluster 1** with the word “*in vitro* meat” is related to the process of artificial meat production, while **Cluster 2** with the word “clean meat” is more related to the challenges and advantages of *in vitro* meat production. **Cluster 3** around the word “cultured meat” describes consumers' acceptance. **Cluster 4** is more related to sustainability and environmental issues for meat in general (Figure 3).

Clusters were obtained using the VOSviewer software, which constructs bibliometric maps of co-occurrence keywords (van Eck and Waltman, 2010), with a minimum of one article with two terms in this case. Keyword co-occurrence analysis is universal in scientometric analysis (Radhakrishnan et al., 2017). It mainly studies the link strengths among co-occurrence keywords in a large variety of literature (Zhao et al., 2019).

Its function is to analyze the internal relationship within an academic field and to reveal the subtopics of research within it.

#### Countries and Institutions Analysis

The research papers related to cultured meat were published mainly by the USA (22.6%), the United Kingdom (14.1%), the Netherlands and Germany (7.6% each), Australia (5.5%), France, and New Zealand (4.0% each), plus other countries (Table 3). The major institutions or local campuses are: INRAE-VetAgro Sup-Clermont University in France and Wageningen University Research in the Netherlands (10 and 9 articles, respectively), whereas publications dealing with cultured meat were published from more diverse groups of institutions in the case of other countries (Table 3).

**Table 3:** Major countries from which articles related to cultured meat were published.

**Countries**	**Number of publications**	**Major institutions/locations**	**Number of publications**
USA	74	University of California system Arizona State University Good Food Institute	7 6 6
United Kingdom	46	University of Bath University of Oxford Brunel University	8 8 5
Germany	25	Helmholtz Association Karlsruhe Institute of Technology	5 5
The Netherlands	25	Wageningen University Research Maastricht University	9 6
Australia	18	Several institutions or locations	<5 each
France	13	INRAE, University of Auvergne, VetAgroSup	10
New Zealand	13	Massey University	7
Canada	12	Several institutions or locations	<5 each
China	12	Several institutions or locations	<5 each
Italy	11	Several institutions or locations	<5 each
Sweden	11	Several institutions or locations	<5 each
India	10	Sher-e-Kashmir University of Agricultural Sciences and Technology of Kashmir	5
Belgium	9	Ghent University	6

The scientific impact of the published articles is presented in Table 4 by institution according to the number of citations, the citation impact (normalized by scientific category), and the proportion of documents in Q1 (the top 25% journals in one scientific category). The articles with the highest impact are from the University of Oxford and Brunel University, which published articles related to the environmental impact of cultured meat and social issues (consumer attitudes, market issues). Articles from the Universities of Bath and of Ghent also have high impacts and also concern consumer behaviors. Wageningen University and French institutions published articles which were also related to social issues (food sustainability, meat alternatives, consumer behaviors). It is noteworthy that Maastricht University, which is Prof. Mark Post's (the leading scientist for cultured meat), has published a relatively low number of scientific articles (6) compared to other institutions (Table 3) and has published scientific papers mainly related to technical issues but with a relatively lower impact compared to other institutions (Table 4).

**Table 4:** Impacts of scientific articles dealing with cultured meat by institution, which published them.

**Organization**	**No. publications**	**Times cited**	**Category normalized citation impact**	**% Documents in Q1 journals**
INRAE	10	239	2.06	29
Wageningen University and Research	9	154	2.70	57
Universite Clermont Auvergne and Associes	8	188	2.17	40
University of Bath	8	119	3.30	100
University of Oxford	8	406	4.53	57
Massey University	7	112	1.92	33
University of California System	7	241	2.08	57
Arizona State University	6	66	1.15	25
Ghent University	6	162	3.37	50
VetAgro Sup	6	92	1.74	25
Maastricht University	5	121	0.91	50
ETH Zurich	5	131	3.96	67
Karlsruhe Institute of Technology	5	40	1.71	75
University of Wisconsin System	5	58	1.34	40
University of London	5	209	1.87	67
Brunel University	5	57	4.52	80
Helmholtz Association	5	40	1.71	75

*Source InCites Clarivate Analytics (InCites dataset updated March 26, 2020. Includes Web of Science (WoS) content indexed through February 29, 2020)*.

#### Journals Network

The major scientific journals, in which articles dealing with cultured meat were published, are journals specialized in meat science [such as *Fleischwirtschaft* (for meat industry), which is the German meat science journal (13 papers); and *Meat Science* (12 papers), which is the internationally renowned scientific journal for meat qualities researchers]. In addition, other journals focusing on social science have published a significant number of papers related to ethics or consumer perception, such as *Journal of Agricultural Environmental Ethics and Appetite* (10 papers each). The *Journal of Integrative Agriculture* (from China) also published a special issue on cultured meat in 2015 with 10 articles.

Seven scientific papers were classified as highly cited papers, but none of them is directly related to *in vitro* meat. They are dealing with food, protein, and meat consumption in general in relation to environmental issues or sustainability, and artificial meat is mentioned as one solution among others.

### Articles Dealing With Cultured Meat in Mainstream Media

#### Time Distribution and Keywords Distribution

**Figure 4:**
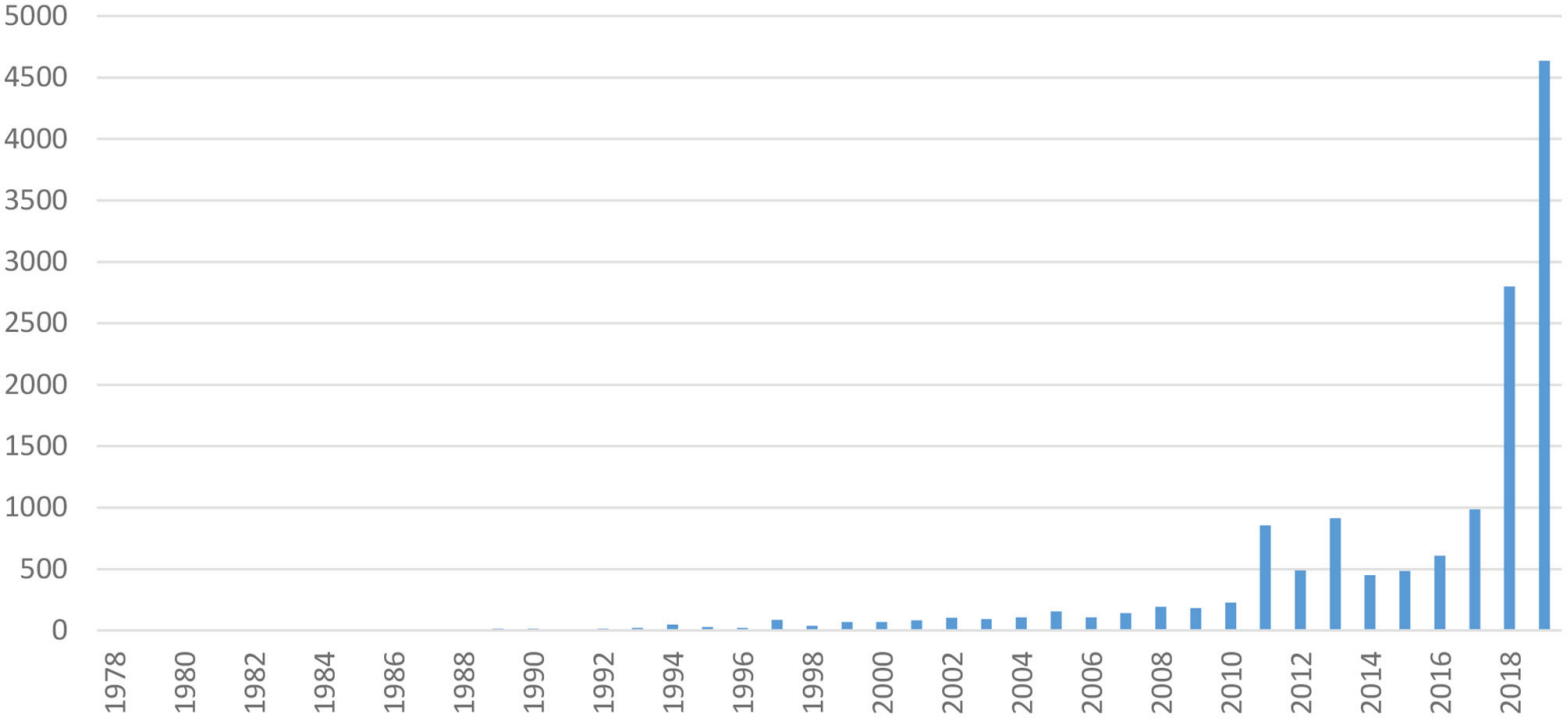
Number of articles dealing with cultured meat and recorded per year in the Factiva bibliometric database.

In the international media, 12,900 press articles dealing with artificial meat have been found through public databases. The evolution of the number of occurrences increased almost exponentially between 1995 and 2019, with a peak of occurrence in 2013 (with 915 articles), particularly after the presentation of the first *in vitro* hamburger by Mark Post in 2013. The “publicity” made at that time by Mark Post was widely reported in the media. The year 2019 alone accounts for more than 36% of publications on the subject with 4,688 articles (and 22% for the year 2018 with 2,801 publications) (Figure 4).

**Figure 5:**
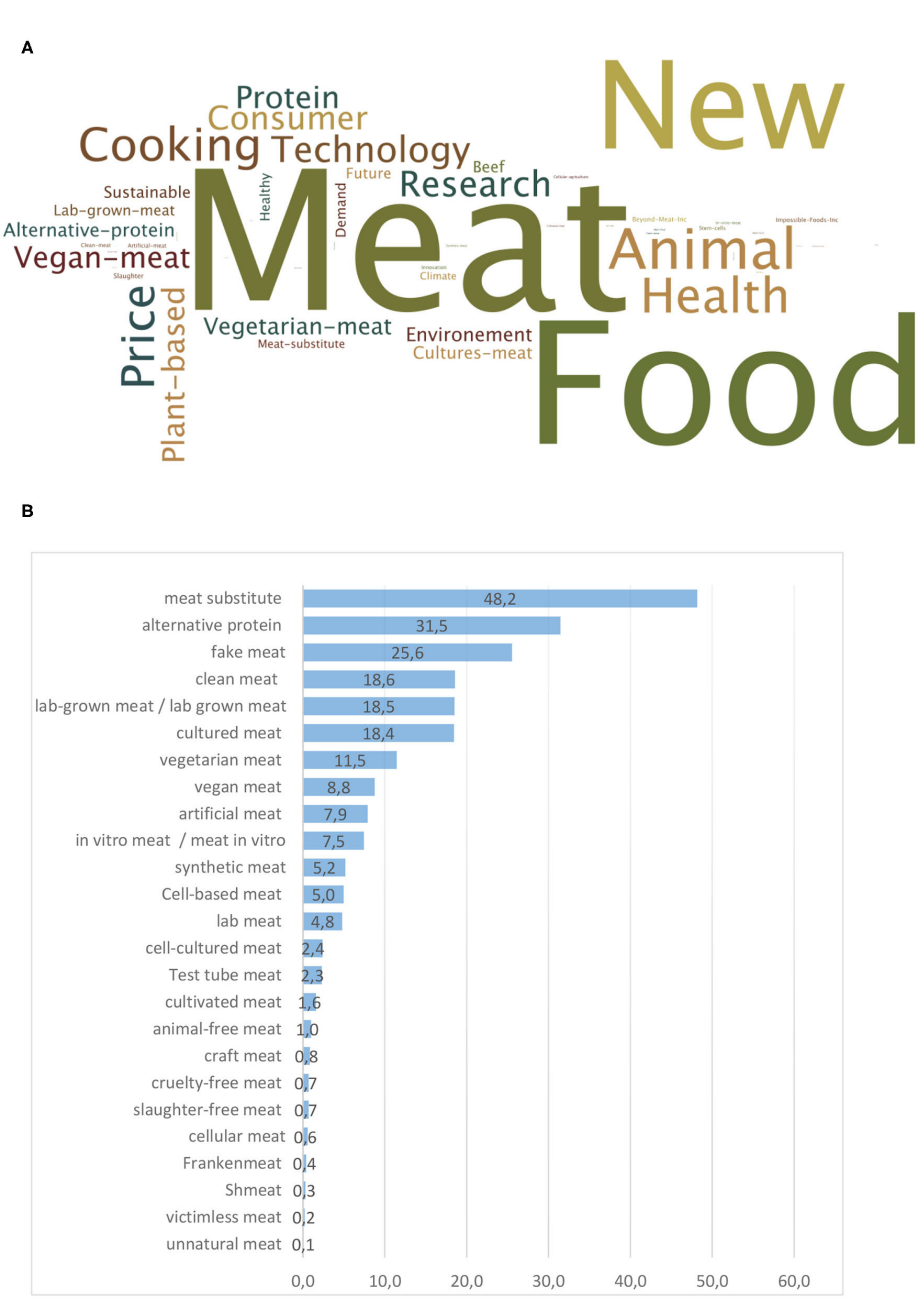
Major keywords in articles from Factiva dealing with cultured meat **(A)** and wording recorded per year **(B)** used in mainstream media to design the *in vitro* meat (occurrence of each wording expressed in percentage of the total, i.e., 12,900 articles).

Predominant keywords are “meat” and to a lesser extent “food,” which might be interpreted by the fact that “cultured meat” is presented as a new type of meat or a novel food (Figure 5A). It is interesting to note that different keywords are sometimes associated in the same publication. However, the predominant wording for this novel food is “meat substitute” (6,213 occurrences) and to a lesser extent “alternative protein” (4,059 occurrences), “fake meat” (3,296 occurrences), “clean meat” (2,396 occurrences), lab-grown meat (2,387 occurrences), and “cultured meat” (2,380 occurrences) (Figure 5B).

#### Analysis by Countries and Institutions

Most articles come from the American press (3,746 articles: 18.3%), United Kingdom (2,199 articles; 17.0%), Australia (880 articles; 6.8%), Canada (748 articles, 5.8%), or New Zealand (579 articles; 4.5%). Around 5.8% of press articles come from China (742 articles), and it is interesting to note that the Netherlands, Mark Post's country and his company Mosa Meat, counts 235 press articles, or 1.8% only of the total (Table 5).

**Table 5:** Number and proportions of articles published in mainstream media according to the country and the language used.

**Countries**	**Number of publications**	**Languages**
USA	3,746 (18.3%)	
UK	2,199 (17.0%)	12,115 out of 12,900 publications (93.9%) are written in English
Australia	880 (6.8%)	
Canada	748 (5.8%)	
New Zealand	579 (4.5%)	
The Netherlands	235 (1.8%)	46 out of 903 publications from China are written in Chinese
China	742 (5.8%)	
Other countries	3,771 (43.0%)	

For the overwhelming majority of articles coming from English-speaking countries, it is not astonishing that 93.9% of those were written in English (12,115 articles) and to a much lower extent in German (428 articles, 4.5%), Chinese (92 articles, 1.0%), French (57 articles, 0.6%), Spanish (40 articles, 0.4%), Italian (39 articles, 0.4%), or Portuguese (30 articles, 0.3%) (Table 5).

About 1,122 articles (9%) were published in international financial newspapers such as Dow Jones Newswires (subsidiary of News Corporation publishing financial information), *The Wall Street Journal* and Barron's magazine, *William Reed Business Media* or *Financial Times*. The articles were also found in well-known newspaper titles such as *The Telegraph, The Guardian, The Times*.

Nevertheless, most of the articles (73%) were published in mainstream media (*PR Newswire, The Times, The Telegraph, The Guardian, The New York Times, Daily Mail*, etc.). It is also interesting to note that 5% of these articles were published in medical (*NewsRx* Medical Newsletter, etc.) or cooking journals (*Food Weekly News*, etc.) (Table 6).

**Table 6:** Number of articles published in mainstream media about cultured meat by press title.

**Journals**	**Number of publications**
*Dow Jones Newswires* (USA)	540
*The Telegraph* (UK)	213
*The Guardian* (UK)	210
*The Times* (UK)	208
*PR Newswire* (USA)	199
*The Wall Street Journal* (USA)	197
*Financial Times* (UK)	195
*William Reed Business Media* (UK)	190
*The New York Times* (USA)	190
*UWire (University Wire)* (USA)	177
*Daily Mail* (UK)	170
*The Independent* (UK)	149
*Postmedia Breaking News* (Canada)	145
*NewsRx Medical Newsletter* (USA)	137

Among the 9,543 articles, respectively, 982 and 443 deal with the theme of “vegetable meats” developed, respectively, by the start-ups Beyond Meat and Impossible Food (Table 7). These plant-based meat producers are the focus of 11% of the articles.

**Table 7:** Number and proportions of articles published in public media about cultured meat by firm or organization.

**Firms or organizations**	**Number of publications**
Beyond Meat Incorporated	982
Impossible Foods Inc.	443
Tyson Foods Inc.	187
Agence sanitaire de sécurité alimentaire	107
United States Department of Agriculture	87
McDonald's Corporation	75
People for the Ethical Treatment of Animals	74
Cargill, Inc.	70
National Cattlemen's Beef Association	69
Burger King Worldwide Inc.	47
Centre for Cellular and Molecular Biology	47
Amazon	42
Food and Agricultural Organization of the United Nations	41
Scotland's Rural College	39
European Union	39

Google, Apple, Facebook, Amazon (GAFA) executives who have invested in these companies are also widely cited in the articles. This is notably the case of Bill Gates (Microsoft, 301 articles) who became an Impossible Food and Beyond Meat investor. Convinced by vegetable meats, Bill Gates declared in 2013: “*I couldn't tell the difference between Beyond Meat chicken and real chicken*.” This is also the case of Sergey Brin (Google, 295 articles) or Jeffrey Bezos (Amazon, 42 articles), who have, respectively, invested in Mosa Meat (cellular meat) and NotCo (novel plant-based meat and dairy alternatives) (Table 8).

**Table 8:** Number of articles published in mainstream media about cultured meat mentioning a celebrity.

**Number of publications**	**Leader**	**Details**
301	William (Bill) Gates (USA)	Cofounder with Paul Allen of the company Microsoft
295	Sergey *(Mikhaylovich)* Brin (Russia)	Cofounder with Larry Page of the company Google
195	Ethan Walden Brown (USA)	Founder of Beyond Meat
111	Patrick Brown (USA)	Founder of Impossible Foods Inc.
78	Scott Gottlieb (USA)	American physician and investor who was the 23rd Commissioner of the Food and Drug Administration from 2017 to April 2019
67	Elon Reeve Musk (Canada)	Cofounder of PayPal
65	Bruce Friedrich (USA)	Cofounder of Good Food Institute
61	Ingrid Newkik (UK)	British animal rights activist, President of People for the Ethical Treatment of Animals, commonly known as PETA
53	Justin Whitmore (USA)	Executive Vice President at Tyson Foods
46	Josh Tetrick (USA)	CEO of JUST, Inc., formerly known as Hampton Creek
43	George Ervin Perdue (USA)	Secretary of Agriculture in President D. Trump's office
42	Jeffrey P. Bezos (USA)	President and Chief Executive Officer of Amazon
38	David Lee (USA)	Chief Financial Officer of Impossible Foods
38	Evan Williams (USA)	Cofounder of Twitter, Blogger and Medium

However, it is also possible to retrieve and classify data from the Factiva database by the names of start-ups (or of their managers) that develop cultured meat. As indicated in Table 9, the major start-ups identified in this way were, in the decreasing number of articles they have published, Mosa Meat (Mark Post), Memphis Meat (Uma Valeti, Nicholas Genovese, or Will Clem), Aleph-Farms (Didier Toubia), Vital Meat (Etienne Duthoit), Gourmey (Nicolas Morin-Forest), Modern Meadow (Andras Forgacs), Hampton Creek/Just (Joshua Tetrick), Higher Steaks (Benjmaina Bollgag), IntegriCulture (Yuki Hanyu), or Vow (George Peppou/Tim Nookesmith). In particular, we can see the development of articles mentioning these companies in recent years.

**Table 9:** Number of articles in the press media about the specific start-ups (or the leaders of these start-ups) that develop cultured meat.

**Start-up *Leader***	**2010**	**2011**	**2012**	**2013**	**2014**	**2015**	**2016**	**2017**	**2018**	**2019**	**2020**	**Year company was founded**
Mosa meat						11	69	71	425	425	174	2015
*Mark Post*	61	144	860	1,168	178	197	171	156	451	894	101	
Memphis meat							15	76	90	118	55	2016
*Uma Valeti, Nicholas Genovese, Will Clem*	2	40	24	8	0	1	80	158	100	55	59	
Aleph Farms								0	99	595	160	2017
*Didier Toubia*									59	146	28	
Vital Meat									11	17	7	2018
*Etienne Duthoit*										12	8	
Gourmey									4	6	7	2019
*Nicolas Morin-Forest*									1	9	6	
Modern Meadow		1	88	96	258	132	149	108	120	188	28	2011
*Andras Forgacs*		0	84	80	78	80	26	14	8	15	0	
Hampton Creek/JUST		0	0	0	0	0	0	208	178	100	16	2011
*Joshua Tetrick*								10	8	8	0	
Higher Steaks								0	4	81	6	2017
*Benjamina Bollag*								0	0	9	0	
integriCulture								0	28	48	18	2017
*Yuki Hanyu*								1	7	8	1	
VOW										0	0	2019
*George Peppou, Tim Noakesmith*										9	0	
Shojinmeat Project							1	5	10	2	5	2014
*Yuki Hanyu*								1	7	3	5	
SuperMeat						16	88	83	354	198	39	2015
*Yaakov Nahmias*						25	43	6	21	58	4	
Finless Foods								0	0	0	0	2017
*Mike Seleden and Brian Wyrwas*								2	7	3	0	
IndieBio					4	42	51	60	78	141	26	2014

### Comparison Between Scientific and Written Press Publications

#### Comparison of Scientific and Written Press Publications Across Countries

One way of comparing scientific and press media publications is to study the frequency of keywords used by authors for the designation of cultured meat among those common in both types of articles.

**Figure 6:**
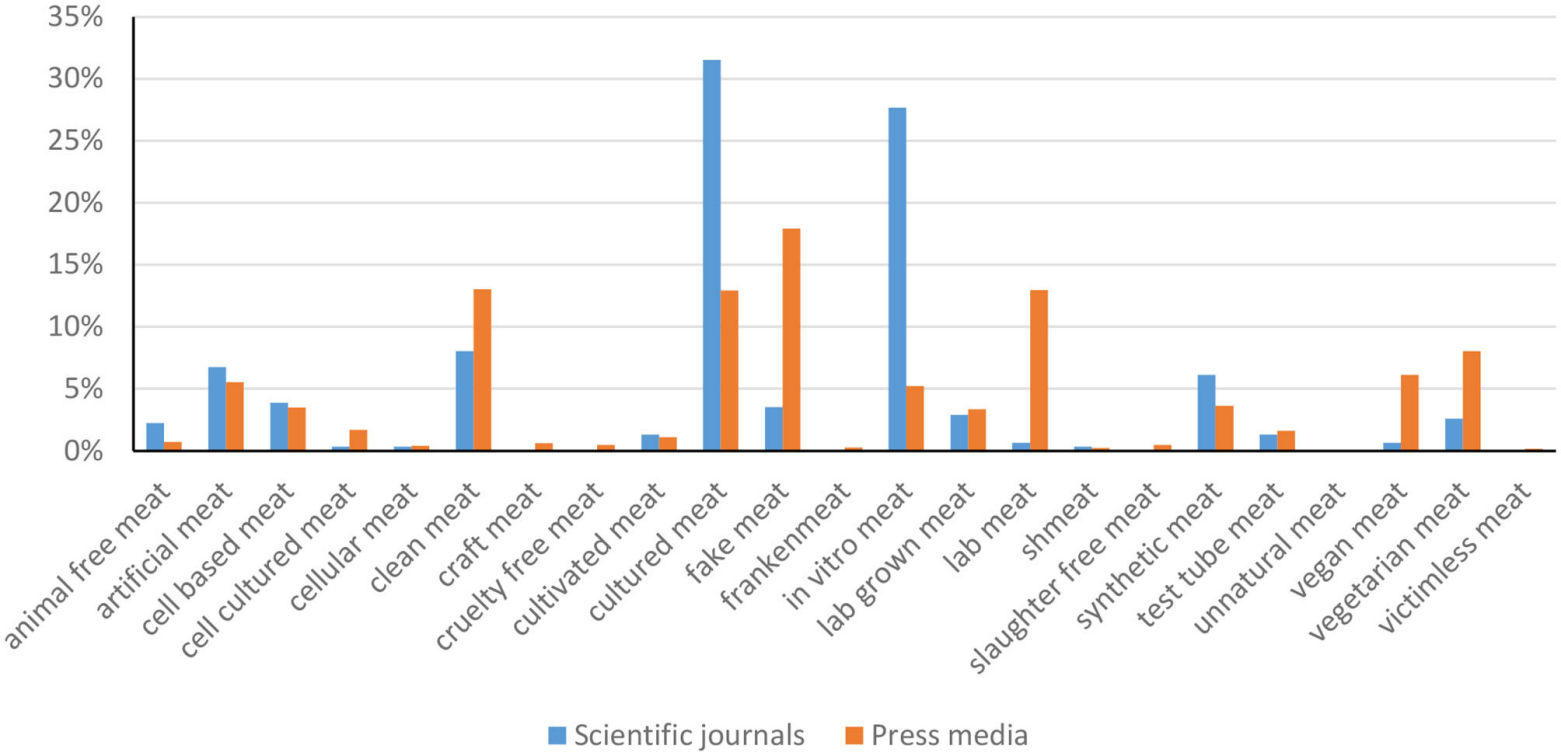
Proportions of keywords in scientific articles and in the written media to designate cultured meat.

As previously observed, the preferred wordings in the scientific literature are “cultured meat” and “*in vitro* meat,” whereas “fake meat,” “cultured meat,” “clean meat,” and “lab meat” (combined with lab-grown meat) are the most frequent wordings used in the written press (Figure 6).

The characteristics for the other articles are roughly the same for both scientific and mainstream articles: they are mainly published in the USA first and in the UK in second place, with a sharp increase from 2017 to 2019. However, the third and fourth countries publishing scientific articles are Germany and the Netherlands for the scientific articles but Australia, Canada, and China for the press articles.

#### Comparison of Scientific and Press Media Publications in China

A specific focus was made on publications in China or in the Chinese language. The reasons are the following: China is the largest country in the world in terms of population, Chinese is the most widely spoken language in the world, the number of press articles about cultured meat has increased by a factor of five between 2018 and 2019, so that China is today the fourth country in the world, i.e., the first non-English-speaking country interested in this new product (after the USA, the UK, and Australia). Furthermore, the concept of “cultured meat” comes from the Western World, and it might be interesting to analyze how it is perceived by such a different culture.

From the WOS database, we found only one scientific publication in Chinese about cultured meat from a total of 12 scientific articles from China. In the Chinese media, 903 press articles dealing with artificial meat have been found through the public database Factiva including 46 in Chinese. A huge increase (by a factor of 5) was observed between 2018 and 2019 (from 83 in 2018 to 400 in 2019). The most frequently used words are “artificial meat,” “cultured meat,” and “*in vitro* meat.” However, Chinese people often use different platforms.

In the CNKI (cnki.net), an academic thesis publication platform, 212 Chinese publications dealing with artificial meat have been found by using all the words related to cultured meat. In addition, before 2019, there were <10 papers published on this subject every year. In 2019, the number of artificial meat-related publications increased to 55. This may be explained by the global trend of increasing worldwide research on artificial meat.

*Baidu Scholar* is a broader publication search platform than CNKI, which can gather publications from multiple websites. From this platform, we found a total of 496 scientific and press articles dealing with cultured meat written in Chinese by Chinese authors. Most of these publications are pieces of review literature aimed to introduce the concept of cultured meat to the general public. In addition, there are also some rigorous pieces of review literature aimed at elaborating the most cutting-edge technologies of artificial meat in the current world. Their aims are, for example, to demonstrate the application of cell culture techniques to cultured meat or to analyze the progress of patent applications related to artificial meat technology all over the world. These elements are expected to provide a reference for the implementation of large-scale production of artificial meat in China.

It is therefore obvious that Chinese academics have a strong interest in research on artificial meat, and there will be more attention on artificial meat with the vegetarian beef and pork products served by Starbucks® in China since April 2020. Although no Chinese original research publications on *in vitro* meat have been found by using Chinese academic websites, original research publications in English on artificial meat from China can be found by using overseas academic websites, such as Google Scholar. This may be due to that the majority of Chinese scientists prefer international peer-reviewed papers and publish work in international platforms. For example, a Chinese team from Jiangnan University designed a large-scale airlift reactor for cultured meat manufacturing, allowing to produce, with a single 300 m^2^ reactor, cultured meat for 75,000 people. On the other hand, Nanjing Agricultural University announced in 2019 that the first cultured meat developed from pig muscle stem cells in China had been produced by a Chinese scientist and his team.

Due to thousands of years of vegetarian diet history, vegetarian meat has a large market in China with a high acceptance by Chinese consumers. In China, artificial meat and vegetarian meat are clearly two different concepts. Chinese publications about vegetarian meat mainly refer to the use of soybean protein as the main ingredient. Vegetarian meat has a large market in China due to the long history of vegetarian diet culture of Chinese people. After searching for vegetarian meat on *Baidu scholar*, 396 publications can be found about patented works on vegetarian food recipes, which has no relationship with cell-tissue engineering. Besides, some pieces of review literature can also be found, such as discussions about the current problems and future development of vegetarian protein meat.

The development of the artificial meat in Western countries has always attracted the attention of Chinese researchers. On *Baidu Scholar*, a number of Chinese publications discussed the development of artificial meat in Western countries, mainly in the United States (79 publications) and in Europe (especially the United Kingdom and the Netherlands with, respectively, 13 and 34 publications). There were also some articles/reports discussing the potential acceptance of artificial meat from America by Chinese consumers.

## Discussion

### Cultured Meat Is an Emerging Topic, Especially in the USA and the UK

Gathering all publications dealing with the same subject, either from scientific journals or from the written press, is never accurate because it depends on the keywords taken into account and on the databases. In our specific case, the same keywords were used for searching both the scientific and the public databases.

Taking into account the small size of the bibliographic corpus, it is likely that we gathered most of the scientific papers dealing with cultured meat by using more than 20 keywords since the number of articles is roughly the same from the two well-known and widely used databases: ISI Web of Science and Scopus (327 and 309, respectively). For the written press, being exhaustive is always a greater challenge due to the diversity of article types, languages, countries of origin, etc. Nevertheless, in both cases, we observed the same trends: the publications are mainly from the USA and the UK, and the number of articles has increased from 2013 and especially from 2017 onward, confirming previously observed trends (Fernandes et al., 2019).

It might be surprising that the media coverage is more or less parallel to the publications of scientific articles. Indeed, public awareness of scientific achievements often appears after a delay depending on the global interest of the media for the subject. In this specific case, there is no delay and even a high ratio of articles in the written press by scientific articles (roughly 30) compared to other subjects such as “meat” with a ratio of 16 only or “cultured cells” with a ratio of roughly one (data not shown). We can thus hypothesize that this is neither the technique *per se* nor the meat subject which is attractive but the combination of both, i.e., the idea to provide new types of meat for the future in a context of anxiety for food security in the future (Gilland, 2002). In addition to that, advocates of artificial meat are very active in the written press since the highly publicized tasting of a cultured beef hamburger on August 5, 2013, in London. The most active countries in terms of publishing scientific articles are mainly the USA and the UK, but also Germany (with many scientific articles in German), the Netherlands, Australia, France, New Zealand, and Canada. However, the Western media, particularly in the USA, the UK (which are also very active in the press media), and Canada, have been perceived to give a biased picture of cultured meat (Goodwin and Shoulders, 2013; Hopkins, 2015).

On the other hand, a huge country like China does not publish so many scientific articles, but in proportion, much more articles in the press media. Most of them are pieces of review literature, which mainly aim to describe the current trend of artificial meat in China and in the whole world. These elements are expected to provide information to rationalize large-scale production of artificial meat in China, a country which is traditionally more oriented toward vegetarian meat.

### The Wording Is Important

It is widely acknowledged that the name given to any object or process can affect subsequent evaluations and feelings about it. In this way, different names were proposed for cultured meat, with different consequences on consumer attitude. They include “*in vitro* meat,” “clean meat,” “cultured meat,” “lab-grown meat,” “synthetic meat,” and other names (Bryant and Barnett, 2019; Bryant C. J. et al., 2019; Ong et al., 2020).

The wordings “fake meat” or “lab meat” are more frequently used in the written press. On the other hand, scientific authors prefer “cultured meat” and “*in vitro* meat.” The latter may reflect the necessity to notify the general public that cultured meat is produced within research labs, which is obvious for scientists. One other interpretation is the fact that popular media use less technical words for a better understanding by readers. Moreover, scientists tend to describe facts without any emotion or judgment, particularly with a novel technology. Maybe this is not the case with a part of the mainstream media, which use terms like “fake” more often. Another explanation is that the term “fake meat” is not exclusively used for *in vitro* meat. Indeed, “fake meat” may also refer to a plant-based product that generally looks and tastes like meat, and this may increase the use of this word particularly in the written press artificially. In scientific literature, the term “fake meat” is mainly used in editorial material (70% of its use), which is not representative of scientific peer-reviewed papers.

Furthermore, the wording “fake meat” could discourage consumers, with possible negative connotations. In fact, the lack of consumer acceptance could be a major barrier to the introduction of cultured meat in the market (Siegrist et al., 2018; Ong et al., 2020) and how the product is framed is of paramount importance for its acceptance by consumers. “Lab-grown meat” is apparently not favorable for high acceptance, whereas “clean meat” is more favorable (Bryant and Barnett, 2019). Otherwise, some authors (Asioli et al., 2018) have demonstrated that consumers tend to strongly reject the name “*in vitro* meat.” Moreover, the term “cultured” is less disliked than the terms “artificial” and “lab-grown” (Asioli et al., 2018). This is confirmed by the study by Siegrist et al. (2018), which concluded that consumers have a low level of acceptance of cultured meat because it is perceived as unnatural. Bryant C. et al. (2019) and Siegrist and Sütterlin (2017) argued that higher acceptance may be favored by less technical descriptions of cultured meat. This may be explained by the fact that the process for “ultra-processed foods” is associated with something scientific and unnatural and, therefore, negatively affects the product's image. In reality, consumers seem to dislike unnatural food. A recent study confirmed that German consumers, despite recognizing the potential ethical advantages of cultured meat, consider themselves to be only moderately prepared to accept cultured meat due to its unnatural status (Weinrich et al., 2020).

### The Issues Around Cultured Meat Are Important

Technical issues about cultured meat still represent challenges, including for advocates of cultured meat. For non-convinced scientists, cultured meat is already obsolete since progress in competing meat substitutes (such as plant-based meat alternatives) is huge, some of these products being already commercialized unlike cultured meat (Warner, 2019). However, the scientific publications with the highest impact are generally not those about technical issues (as those from M. Post) but those from a limited number of researchers from the universities of Bath, Oxford, or Ghent, which are more related to social sciences (such as acceptance by consumers) [e.g., van der Weele et al. (2019)] and/or environmental issues [such as Tuomisto and de Mattos (2011)]. Indeed, in some countries, such as the Netherlands, France, and New-Zealand, scientific articles are published by one or two groups only, discussing the advantages and limitations of cultured meat. In the Netherlands, the two active groups are Wageningen University Research and Maastricht University (the former is very active in social science) [e.g., van der Weele et al. (2019)], while the latter is the institution where M. Post is very active in tissue engineering [e.g., Post (2012)].

These issues about cultured meat have been evidenced by cluster 4 of the cluster analysis of published scientific articles. This cluster is not restricted to cultured meat but considers all issues related to meat production such as food supply by sustainable productions including meat substitutes and any type of alternatives to meat (Bonny et al., 2017). One important issue, which is a cluster *per se*, is the potential benefits of artificial meat in terms of health and climate protection encapsulated in the concept of “clean meat.” Cultured meat is thus an option for consumers and citizens who do not want to stop eating meat but who are willing to decrease the potential disadvantages of meat production and consumption.

### New Consumption Behavior

Flexitarianism has been developing in recent years and was designated as the “food trend of the year 2017” (Dagevos and Reinders, 2018). The same year, a similar trend called “the reducetarian” appeared (Kateman, 2017). This trend toward lower meat consumption is thus observed in many countries. It is sustained with various issues related to meat consumption (such as ethics, the environment, health, etc.), independent of economic reasons.

Although it is unknown how many flexitarians already existed in the second half of the previous century, scholarly attention to meat reduction practices in the last few years provides evidence that flexitarianism constitutes a genuine food consumer segment (Dagevos and Reinders, 2018).

This evolution can be seen in the terms commonly found in the topics covered by press articles. The frequency of wordings related to “alternative method” of meat production (such as “meat substitute,” “alternative protein,” “vegetarian meat,” and “vegan meat”) is also not surprising. It can thus be hypothesized that a sizable share of press articles targeted readers whose consumption behavior has evolved toward a lower consumption of meat and a higher consumption of plant-based meat substitutes in the last few years.

Many authors agree that diets for which most calories come from plant sources while limiting or avoiding animal sources are more sustainable, healthier, and alleviate animal suffering (Sabaté, 2003; De Boer and Aiking, 2011; Graça et al., 2015). In spite of these benefits, consumers in Western societies do not seem willing to reduce their meat consumption (Latvala et al., 2012; Schösler et al., 2012). In this context, cultured meat is possibly a viable alternative (which is presented as such in the press) all the more as the most promising pathways to encourage large-scale shifts toward less meat-based diets are likely the ones that do not challenge existing meal formats and hierarchies, in which meat has a central role (Schösler et al., 2012).

### Drivers of Consumer Acceptance of Cultured Meat

During the introduction of this technology to the public, it became clear that public acceptance was not immediate and perhaps not obvious. The theoretical framework on rejection of novel and unfamiliar foods was laid down by Rozin and Fallon (1980).

Verbeke et al. (2015) indicate that only 10% of consumers would be really opposed to *in vitro* meat, the vast majority having a rather hesitant attitude. Other works have highlighted the importance of the perception of “ultra-processed foods” such as *in vitro* meat, which results in less consent to buy or to eat this product, contrary to claims related to its societal benefits or to its similarity to conventional meat (Bryant and Dillard, 2019; Ong et al., 2020). A recent review has highlighted that the main motivations for acceptance of meat substitutes are criteria related to good health and meeting the nutritional needs of consumers rather than collective values (such as environmental protection or animal welfare) (Chriki and Hocquette, 2020).

However, consumer acceptance is likely to increase when consumers become more familiar with the concept of cultured meat, as they are bound to become increasingly reassured if the product becomes authorized, accessible, and available (Bryant and Barnett, 2019), and as its name becomes more attractive (Ong et al., 2020).

Thus, using quite “positive” wordings (such as “meat substitute,” “alternative protein,” “vegetarian meat,” “vegan meat,” but also “cruelty-free meat,” “animal-free meat,” “victimless meat”) is particularly interesting to consider; indeed, a recent research article (Rolland et al., 2020) has concluded that having positive information improves acceptance and willingness to taste “cultured” meat. According to Grunert et al. (2004), the potential for success of new products can be better exploited by developing products that are solicited and/or requested by consumers. Creating a new expectation around artificial meat is thus a favorable opportunity to enable its development and appropriation by consumers.

## Conclusion

Cultured meat has become an emerging topic in both the scientific and media literature, especially in the last 3 years. It is mainly developing in the USA and the UK, with other countries, such as China observing the trend for potential future applications. The wordings of the scientific literature (mainly “cultured meat,” “*in vitro* meat”) indicate that scientific articles seem to focus, at least initially, mainly on the methods and technical aspects of artificial meat. However, more and more published studies are now focused on advancements, challenges, and potential advantages of cultured meat because most of the technical issues are thought to be solvable at some point in time. Thus, at the present time, the technique seems to be increasingly well-mastered and it no longer seems to be the “rate-limiting point” for the development of artificial meat on a large scale, even if this view is not shared by all scientists. Thus, articles reporting on technical aspects tended in recent months to give way to more general considerations about the health value of artificial meat and its acceptance by consumers, which seem to be a greater concern for them. Through the occurrence of the term “clean meat,” reference to the environment-friendly effects of this technology is also more and more represented in the press and scientific articles. These trends are mainly observed in the written press with has a greater interest for this topic.

## Data Availability Statement

All datasets presented in this study are included in the article/Supplementary Material.

## Author Contributions

SC, M-PE-O, and J-FH contributed equally in the redaction of this paper. WoS analysis was done by DF. JL has done analyses of the Chinese publications.

## Conflict of Interest

The authors declare that the research was conducted in the absence of any commercial or financial relationships that could be construed as a potential conflict of interest.
