# Supplementary material for: Analysis of Scientific and Press Articles Related to Cultured Meat for a Better Understanding of Its Perception
Source: Front Psychol. 2020 Aug 25;11:1845. doi: 10.3389/fpsyg.2020.01845 (PMC7477931; doi:10.3389/fpsyg.2020.01845)
Supplement: Supplementary file 1 [file Data_Sheet_1.PDF]

List of scientific publications related to cultured meat

Weinrich, R; Strack, M; Neugebauer, F (2020) Consumer acceptance of cultured meat in Germany, MEAT SCIENCE, doi:10.1016/j.meatsci.2019.107924 - WOS:000512481600023

Zhang, GQ; Zhao, XR; Li, XL; Du, GC; Zhou, JW; Chen, J (2020) Challenges and possibilities for bio-manufacturing cultured meat, TRENDS IN FOOD SCIENCE & TECHNOLOGY, doi:10.1016/j.tifs.2020.01.026 - WOS:000517848300035

Bodiou, V; Moutsatsou, P; Post, MJ (2020) Microcarriers for Upscaling Cultured Meat Production, FRONTIERS IN NUTRITION, doi:10.3389/fnut.2020.00010 - WOS:000518957700001

Chriki, S; Hocquette, JF (2020) The Myth of Cultured Meat: A Review, FRONTIERS IN NUTRITION, doi:10.3389/fnut.2020.00007 - WOS:000517163200001

Kenigsberg, JA; Zivotofsky, AZ (2020) A Jewish Religious Perspective on Cellular Agriculture, FRONTIERS IN SUSTAINABLE FOOD SYSTEMS, doi:10.3389/fsufs.2019.00128 - WOS:000511332300001

Li, XL; Zhang, GQ; Zhao, XR; Zhou, JW; Du, GC; Chen, J (2020) A conceptual air-lift reactor design for large scale animal cell cultivation in the context of in vitro meat production, CHEMICAL ENGINEERING SCIENCE, doi:10.1016/j.ces.2019.115269 - WOS:000498520300002

Okamoto, Y; Haraguchi, Y; Sawamura, N; Asahi, T; Shimizu, T (2020) Mammalian cell cultivation using nutrients extracted from microalgae, BIOTECHNOLOGY PROGRESS, doi:10.1002/btpr.2941 - WOS:000501480400001

Mohorcich, J; Reese, J (2019) Cell-cultured meat: Lessons from GMO adoption and resistance, APPETITE, doi:10.1016/j.appet.2019.104408 - WOS:000494970500004

Gomez-Luciano, CA; de Aguiar, LK; Vriesekoop, F; Urbano, B (2019) Consumers' willingness to purchase three alternatives to meat proteins in the United Kingdom, Spain, Brazil and the Dominican Republic, FOOD QUALITY AND PREFERENCE, doi:10.1016/j.foodqual.2019.103732 - WOS:000484875100017

Bomgardner, M (2019) FOOD Cultured meat grows in space and on Earth, CHEMICAL & ENGINEERING NEWS, doi: - WOS:000491215700021

Faccio, E; Fovino, LGN (2019) Food Neophobia or Distrust of Novelties? Exploring Consumers' Attitudes toward GMOs, Insects and Cultured Meat, APPLIED SCIENCES-BASEL, doi:10.3390/app9204440 - WOS:000496269400243

Simsa, R; Yuen, J; Stout, A; Rubio, N; Fogelstrand, P; Kaplan, DL (2019) Extracellular Heme Proteins Influence Bovine Myosatellite Cell Proliferation and the Color of Cell-Based Meat, FOODS, doi:10.3390/foods8100521 - WOS:000494272000096

Shaw, E; Iomaire, MM (2019) A comparative analysis of the attitudes of rural and urban consumers towards cultured meat, BRITISH FOOD JOURNAL, doi:10.1108/BFJ-07-2018-0433 - WOS:000474700100008

Bryant, CJ; Anderson, JE; Asher, KE; Green, C; Gasteratos, K (2019) Strategies for overcoming aversion to unnaturalness: The case of clean meat, MEAT SCIENCE, doi:10.1016/j.meatsci.2019.04.004 - WOS:000469159000005

Bryant, C; Dillard, C (2019) The Impact of Framing on Acceptance of Cultured Meat, FRONTIERS IN NUTRITION, doi:10.3389/fnut.2019.00103 - WOS:000473749700001

Bhat, ZF; Morton, JD; Mason, SL; Bekhit, AEA; Bhat, HF (2019) Technological, Regulatory, and Ethical Aspects of In Vitro Meat: A Future Slaughter-Free Harvest, COMPREHENSIVE REVIEWS IN FOOD SCIENCE AND FOOD SAFETY, doi:10.1111/1541-4337.12473 - WOS:000474294600019

van der Weele, C; Feindt, P; van der Goot, AJ; van Mierlo, B; van Boekel, M (2019) Meat alternatives: an integrative comparison, TRENDS IN FOOD SCIENCE & TECHNOLOGY, doi:10.1016/j.tifs.2019.04.018 - WOS:000471083900043

Bryant, CJ; Barnett, JC (2019) What's in a name? Consumer perceptions of in vitro meat under different names, APPETITE, doi:10.1016/j.appet.2019.02.021 - WOS:000464298700011

Gomez-Luciano, CA; Vriesekoop, F; Urbano, B (2019) TOWARDS FOOD SECURITY OF ALTERNATIVE DIETARY PROTEINS: A COMPARISON BETWEEN SPAIN AND THE DOMINICAN REPUBLIC, AMFITEATRU ECONOMIC, doi:10.24818/EA/2019/51/393 - WOS:000465379800009

Wilks, M; Phillips, CJC; Fielding, K; Hornsey, MJ (2019) Testing potential psychological predictors of attitudes towards cultured meat, APPETITE, doi:10.1016/j.appet.2019.01.027 - WOS:000463293900017

Rubio, NR; Fish, KD; Trimmer, BA; Kaplan, DL (2019) Possibilities for Engineered Insect Tissue as a Food Source, FRONTIERS IN SUSTAINABLE FOOD SYSTEMS, doi:10.3389/fsufs.2019.00024 - WOS:000501895200001

Mancini, MC; Antonioli, F (2019) Exploring consumers' attitude towards cultured meat in Italy, MEAT SCIENCE, doi:10.1016/j.meatsci.2018.12.014 - WOS:000458711200013

Mouat, MJ; Prince, R; Roche, MM (2019) Making Value Out of Ethics: The Emerging Economic Geography of Lab-grown Meat and Other Animal-free Food Products, ECONOMIC GEOGRAPHY, doi:10.1080/00130095.2018.1508994 - WOS:000461029600002

Ikeda, K; Takeuchi, S (2019) Anchorage-dependent cell expansion in fiber-shaped microcarrier aggregates, BIOTECHNOLOGY PROGRESS, doi:10.1002/btpr.2755 - WOS:000467996300013

Bryant, C; Szejda, K; Parekh, N; Desphande, V; Tse, B (2019) A Survey of Consumer Perceptions of Plant-Based and Clean Meat in the USA, India, and China, FRONTIERS IN SUSTAINABLE FOOD SYSTEMS, doi:10.3389/fsufs.2019.00011 - WOS:000505575700001

Lynch, J; Pierrehumbert, R (2019) Climate Impacts of Cultured Meat and Beef Cattle, FRONTIERS IN SUSTAINABLE FOOD SYSTEMS, doi:10.3389/fsufs.2019.00005 - WOS:000501891400001

Rubio, NR; Fish, KD; Trimmer, BA; Kaplan, DL (2019) In Vitro Insect Muscle for Tissue Engineering Applications, ACS BIOMATERIALS SCIENCE & ENGINEERING, doi:10.1021/acsbiomaterials.8b01261 - WOS:000458937900062

Jonsson, E; Linne, T; McCrow-Young, A (2019) Many Meats and Many Milks? The Ontological Politics of a Proposed Post-animal Revolution, SCIENCE AS CULTURE, doi:10.1080/09505431.2018.1544232 - WOS:000458405300004

Flachowsky, G; Sudekum, KH; Meyer, U (2019) Protein of Animal Origin: Are there Alternatives?, ZUCHTUNGSKUNDE, doi: - WOS:000474768000002

Loveday, SM (2019) Food Proteins: Technological, Nutritional, and Sustainability Attributes of Traditional and Emerging Proteins, ANNUAL REVIEW OF FOOD SCIENCE AND TECHNOLOGY, VOL 10, doi:10.1146/annurev-food-032818-121128 - WOS:000463604800014

Tuomisto, HL (2019) The eco-friendly burger Could cultured meat improve the environmental sustainability of meat products?, EMBO REPORTS, doi:10.15252/embr.201847395 - WOS:000459024800015

Sebo, J (2018) THE ETHICS AND POLITICS OF PLANT-BASED AND CULTURED MEAT, ATELIERS DE L ETHIQUE-THE ETHICS FORUM, doi:10.7202/1055123ar - WOS:000456208500009

Parodi, A; Leip, A; De Boer, IJM; Slegers, PM; Ziegler, F; Temme, EHM; Herrero, M; Tuomisto, H; Valin, H; Van Middelaar, CE; Van Loon, JJA; Van Zanten, HHE (2018) The potential of future foods for sustainable and healthy diets, NATURE SUSTAINABILITY, doi:10.1038/s41893-018-0189-7 - WOS:000453337000016

Sexton, AE (2018) Eating for the post-Anthropocene: Alternative proteins and the biopolitics of edibility, TRANSACTIONS OF THE INSTITUTE OF BRITISH GEOGRAPHERS, doi:10.1111/tran.12253 - WOS:000449935900005

Hamdan, MN; Post, MJ; Ramli, MA; Mustafa, AR (2018) Cultured Meat in Islamic Perspective, JOURNAL OF RELIGION & HEALTH, doi:10.1007/s10943-017-0403-3 - WOS:000446335000012

Brainard, J (2018) Agencies carve up cultured meat, SCIENCE, doi: - WOS:000451609000012

Bryant, C; Barnett, J (2018) Consumer acceptance of cultured meat: A systematic review, MEAT SCIENCE, doi:10.1016/j.meatsci.2018.04.008 - WOS:000446794700002

Stephens, N; Di Silvio, L; Dunsford, I; Ellis, M; Glencross, A; Sexton, A (2018) Bringing cultured meat to market: Technical, socio-political, and regulatory challenges in cellular agriculture, TRENDS IN FOOD SCIENCE & TECHNOLOGY, doi:10.1016/j.tifs.2018.04.010 - WOS:000440960900013

Ding, SJ; Swennen, GNM; Messmer, T; Gagliardi, M; Molin, DGM; Li, CB; Zhou, GH; Post, MJ (2018) Maintaining bovine satellite cells stemness through p38 pathway, SCIENTIFIC REPORTS, doi:10.1038/s41598-018-28746-7 - WOS:000438850900018

Krieger, J; Park, BW; Lambert, CR; Malcuit, C (2018) 3D skeletal muscle fascicle engineering is improved with TGF-beta 1 treatment of myogenic cells and their co-culture with myofibroblasts, PEERJ, doi:10.7717/peerj.4939 - WOS:000438420500001

Stephens, N; King, E; Lyall, C (2018) Blood, meat, and upscaling tissue engineering: Promises, anticipated markets, and performativity in the biomedical and agri-food sectors, BIOSOCIETIES, doi:10.1057/s41292-017-0072-1 - WOS:000449364200003

Murray, A (2018) Meat cultures: Lab-grown meat and the politics of contamination, BIOSOCIETIES, doi:10.1057/s41292-017-0082-z - WOS:000449364200010

Slade, P (2018) If you build it, will they eat it? Consumer preferences for plant-based and cultured meat burgers, APPETITE, doi:10.1016/j.appet.2018.02.030 - WOS:000430777900046

Servick, K (2018) US lawmakers float plan to regulate cultured meat, SCIENCE, doi:10.1126/science.360.6390.695 - WOS:000432473500016

Siegrist, M; Sutterlin, B; Hartmann, C (2018) Perceived naturalness and evoked disgust influence acceptance of cultured meat, MEAT SCIENCE, doi:10.1016/j.meatsci.2018.02.007 - WOS:000429754300029

Specht, EA; Welch, DR; Clayton, EMR; Lagally, CD (2018) Opportunities for applying biomedical production and manufacturing methods to the development of the clean meat industry, BIOCHEMICAL ENGINEERING JOURNAL, doi:10.1016/j.bej.2018.01.015 - WOS:000433266500018

Verbruggen, S; Daan, LN; van Essen, A; Post, MJ (2018) Bovine myoblast cell production in a microcarriers-based system, CYTOTECHNOLOGY, doi:10.1007/s10616-017-0101-8 - WOS:000427591500003

Chauvet, DJ (2018) Should cultured meat be refused in the name of animal dignity?, ETHICAL THEORY AND MORAL PRACTICE, doi:10.1007/s10677-018-9888-4 - WOS:000431954300016

Lupton, D; Turner, B (2018) Food of the Future? Consumer Responses to the Idea of 3D-Printed Meat and Insect-Based Foods, FOOD AND FOODWAYS, doi:10.1080/07409710.2018.1531213 - WOS:000458277900001

Mouat, MJ; Prince, R (2018) Cultured meat and cowless milk: on making markets for animal-free food, JOURNAL OF CULTURAL ECONOMY, doi:10.1080/17530350.2018.1452277 - WOS:000436834100004

Mattick, CS (2018) Cellular agriculture: The coming revolution in food production, BULLETIN OF THE ATOMIC SCIENTISTS, doi:10.1080/00963402.2017.1413059 - WOS:000428630800007

Alexander, P; Brown, C; Arneth, A; Dias, C; Finnigan, J; Moran, D; Rounsevell, MDA (2017) Could consumption of insects, cultured meat or imitation meat reduce global agricultural land use?, GLOBAL FOOD SECURITY-AGRICULTURE POLICY ECONOMICS AND ENVIRONMENT, doi:10.1016/j.gfs.2017.04.001 - WOS:000417152000003

Schuster, E; Wallin, P; Klose, FP; Gold, J; Strom, A (2017) Correlating network structure with functional properties of capillary alginate gels for muscle fiber formation, FOOD HYDROCOLLOIDS, doi:10.1016/j.foodhyd.2017.05.036 - WOS:000405985200022

Warner, RD; Bittner, EP; Ashman, H (2017) What is meat in Australia?, ANIMAL FRONTIERS, doi:10.2527/af.2017.0443 - WOS:000457269700011

Bekker, GA; Tobi, H; Fischer, ARH (2017) Meet meat: An explorative study on meat and cultured meat as seen by Chinese, Ethiopians and Dutch, APPETITE, doi:10.1016/j.appet.2017.03.009 - WOS:000402347800011

Siegrist, M; Sutterlin, B (2017) Importance of perceived naturalness for acceptance of food additives and cultured meat, APPETITE, doi:10.1016/j.appet.2017.03.019 - WOS:000400218300038

Stephens, N; Lewis, J (2017) Doing laboratory ethnography: reflections on method in scientific workplaces, QUALITATIVE RESEARCH, doi:10.1177/1468794116678040 - WOS:000400139300006

Hartmann, C; Siegrist, M (2017) Consumer perception and behaviour regarding sustainable protein consumption: A systematic review, TRENDS IN FOOD SCIENCE & TECHNOLOGY, doi:10.1016/j.tifs.2016.12.006 - WOS:000396959800002

O'Riordan, K; Fotopoulou, A; Stephens, N (2017) The first bite: Imaginaries, promotional publics and the laboratory grown burger, PUBLIC UNDERSTANDING OF SCIENCE, doi:10.1177/0963662516639001 - WOS:000399565500003

Leroy, F; Praet, I (2017) Animal Killing and Postdomestic Meat Production, JOURNAL OF AGRICULTURAL & ENVIRONMENTAL ETHICS, doi:10.1007/s10806-017-9654-y - WOS:000397201100005

Bonny, SPF; Gardner, GE; Pethick, DW; Hocquette, JF (2017) Artificial meat and the future of the meat industry, ANIMAL PRODUCTION SCIENCE, doi:10.1071/AN17307 - WOS:000412097600004

Bekker, GA; Fischer, ARH; Tobi, H; van Trijp, HCM (2017) Explicit and implicit attitude toward an emerging food technology: The case of cultured meat, APPETITE, doi:10.1016/j.appet.2016.10.002 - WOS:000390622000028

Jonsson, E (2017) On Resurrected Nuggets and Sphincter Windows: Cultured Meat, Art, and the Discursive Subsumption of Nature, SOCIETY & NATURAL RESOURCES, doi:10.1080/08941920.2017.1304599 - WOS:000402012200005

Arshad, MS; Javed, M; Sohaib, M; Saeed, F; Imran, A; Amjad, Z (2017) Tissue engineering approaches to develop cultured meat from cells: A mini review, COGENT FOOD & AGRICULTURE, doi:10.1080/23311932.2017.1320814 - WOS:000461120500077

Post, MJ; Hocquette, JF (2017) New Sources of Animal Proteins: Cultured Meat, NEW ASPECTS OF MEAT QUALITY: FROM GENES TO ETHICS, doi:10.1016/B978-0-08-100593-4.00017-5 - WOS:000467540100016

Hamdan, MN; Ramli, MA (2016) Cultured Meat in Islamic Perspective: An Analysis to the Use of ESCs as Source of Stem Cell, GLOBAL JOURNAL AL-THAQAFAH, doi: - WOS:000414904100010

Hocquette, JF (2016) Is in vitro meat the solution for the future?, MEAT SCIENCE, doi:10.1016/j.meatsci.2016.04.036 - WOS:000381542700019

Stephens, N; Ruivenkamp, M (2016) Promise and Ontological Ambiguity in the In vitro Meat Imagescape: From Laboratory Myotubes to the Cultured Burger, SCIENCE AS CULTURE, doi:10.1080/09505431.2016.1171836 - WOS:000383252800002

Sharma, S; Thind, SS; Kaur, A (2015) In vitro meat production system: why and how?, JOURNAL OF FOOD SCIENCE AND TECHNOLOGY-MYSORE, doi:10.1007/s13197-015-1972-3 - WOS:000365094600004

Mattick, CS; Landis, AE; Allenby, BR; Genovese, NJ (2015) Anticipatory Life Cycle Analysis of In Vitro Biomass Cultivation for Cultured Meat Production in the United States, ENVIRONMENTAL SCIENCE & TECHNOLOGY, doi:10.1021/acs.est.5b01614 - WOS:000362629100079

Laestadius, L (2015) Public Perceptions of the Ethics of In-vitro Meat: Determining an Appropriate Course of Action, JOURNAL OF AGRICULTURAL & ENVIRONMENTAL ETHICS, doi:10.1007/s10806-015-9573-8 - WOS:000361997100013

Zwart, H (2015) Tainted Food and the Icarus Complex: Psychoanalysing Consumer Discontent from Oyster Middens to Oryx and Crake, JOURNAL OF AGRICULTURAL & ENVIRONMENTAL ETHICS, doi:10.1007/s10806-015-9530-6 - WOS:000352209100004

Verbeke, W; Marcu, A; Rutsaert, P; Gaspar, R; Seibt, B; Fletcher, D; Barnett, J (2015) 'Would you eat cultured meat?': Consumers' reactions and attitude formation in Belgium, Portugal and the United Kingdom, MEAT SCIENCE, doi:10.1016/j.meatsci.2014.11.013 - WOS:000349428500008

Dilworth, T; McGregor, A (2015) Moral Steaks? Ethical Discourses of In Vitro Meat in Academia and Australia, JOURNAL OF AGRICULTURAL & ENVIRONMENTAL ETHICS, doi:10.1007/s10806-014-9522-y - WOS:000347889400006

Roberts, RM; Yuan, Y; Genovese, N; Ezashi, T (2015) Livestock Models for Exploiting the Promise of Pluripotent Stem Cells, ILAR JOURNAL, doi:10.1093/ilar/ilv005 - WOS:000356095500007

Kadim, IT; Mahgoub, O; Baqir, S; Faye, B; Purchas, R (2015) Cultured meat from muscle stem cells: A review of challenges and prospects, JOURNAL OF INTEGRATIVE AGRICULTURE, doi:10.1016/S2095-3119(14)60881-9 - WOS:000349516100005

Sun, ZC; Yu, QL; Han, L (2015) The environmental prospects of cultured meat in China, JOURNAL OF INTEGRATIVE AGRICULTURE, doi:10.1016/S2095-3119(14)60891-1 - WOS:000349516100006

Mattick, CS; Landis, AE; Allenby, BR (2015) A case for systemic environmental analysis of cultured meat, JOURNAL OF INTEGRATIVE AGRICULTURE, doi:10.1016/S2095-3119(14)60885-6 - WOS:000349516100008

Bonny, SPF; Gardner, GE; Pethick, DW; Hocquettez, JF (2015) What is artificial meat and what does it mean for the future of the meat industry?, JOURNAL OF INTEGRATIVE AGRICULTURE, doi:10.1016/S2095-3119(14)60888-1 - WOS:000349516100009

Hopkins, PD (2015) Cultured meat in western media: The disproportionate coverage of vegetarian reactions, demographic realities, and implications for cultured meat marketing, JOURNAL OF INTEGRATIVE AGRICULTURE, doi:10.1016/S2095-3119(14)60883-2 - WOS:000349516100010

Bhat, ZF; Kumar, S; Fayaz, H (2015) In vitro meat production: Challenges and benefits over conventional meat production, JOURNAL OF INTEGRATIVE AGRICULTURE, doi:10.1016/S2095-3119(14)60887-X - WOS:000349516100007

Verbeke, W; Sans, P; Van Loo, EJ (2015) Challenges and prospects for consumer acceptance of cultured meat, JOURNAL OF INTEGRATIVE AGRICULTURE, doi:10.1016/S2095-3119(14)60884-4 - WOS:000349516100012

Winiwarter, W; Leip, A; Tuomisto, HL; Haastrup, P (2014) A European perspective of innovations towards mitigation of nitrogen-related greenhouse gases, CURRENT OPINION IN ENVIRONMENTAL SUSTAINABILITY, doi:10.1016/j.cosust.2014.07.006 - WOS:000345230500005

van der Weele, C; Tramper, J (2014) Cultured meat: every village its own factory?, TRENDS IN BIOTECHNOLOGY, doi:10.1016/j.tibtech.2014.04.009 - WOS:000336879300002

Buscemi, F (2014) From killing cows to culturing meat, BRITISH FOOD JOURNAL, doi:10.1108/BFJ-11-2012-0288 - WOS:000341656900005

Bhat, ZF; Bhat, H; Pathak, V (2014) Prospects for In Vitro Cultured Meat - A Future Harvest, PRINCIPLES OF TISSUE ENGINEERING, 4TH EDITION, doi:10.1016/B978-0-12-398358-9.00079-3 - WOS:000427825200084

Goodwin, JN; Shoulders, CW (2013) The future of meat: A qualitative analysis of cultured meat media coverage, MEAT SCIENCE, doi:10.1016/j.meatsci.2013.05.027 - WOS:000322558100001

Coghlan, A (2013) One burger doesn't make a cultured meat industry, NEW SCIENTIST, doi: - WOS:000323096100005

Welin, S (2013) Introducing the new meat. Problems and prospects, ETIKK I PRAKSIS, doi: - WOS:000320154700003

Post, MJ (2012) Cultured meat from stem cells: Challenges and prospects, MEAT SCIENCE, doi:10.1016/j.meatsci.2012.04.008 - WOS:000306881800018

Forgacs, G; Jakab, K; Marga, F (2012) Cultured meat by self-assembly, JOURNAL OF TISSUE ENGINEERING AND REGENERATIVE MEDICINE, doi: - WOS:000308313003203

Wallin, P; Hoglund, K; Wildt-Persson, K; Gold, J (2012) Skeletal myoblast differentiation on starch microspheres for the development of cultured meat, JOURNAL OF TISSUE ENGINEERING AND REGENERATIVE MEDICINE, doi: - WOS:000308313003202

Mattick, CS; Allenby, BR (2012) Cultured Meat: The Systemic Implications of an Emerging Technology, 2012 IEEE INTERNATIONAL SYMPOSIUM ON SUSTAINABLE SYSTEMS AND TECHNOLOGY (ISSST), doi: - WOS:000309221700045

Tuomisto, HL; de Mattos, MJT (2011) Environmental Impacts of Cultured Meat Production, ENVIRONMENTAL SCIENCE & TECHNOLOGY, doi:10.1021/es200130u - WOS:000292850200029

Bhat, ZF; Fayaz, H (2011) Prospectus of cultured meat-advancing meat alternatives, JOURNAL OF FOOD SCIENCE AND TECHNOLOGY-MYSORE, doi:10.1007/s13197-010-0198-7 - WOS:000287842500001

Hopkins, PD; Dacey, A (2008) Vegetarian Meat: Could Technology Save Animals and Satisfy Meat Eaters?, JOURNAL OF AGRICULTURAL & ENVIRONMENTAL ETHICS, doi:10.1007/s10806-008-9110-0 - WOS:000260469900005

Edelman, PD; McFarland, DC; Mironov, VA; Matheny, JG (2005) In vitro-cultured meat production, TISSUE ENGINEERING, doi:10.1089/ten.2005.11.659 - WOS:000230320600001

in vitro meat :

Warner, RD (2019) Review: Analysis of the process and drivers for cellular meat production, ANIMAL, doi:10.1017/S1751731119001897 - WOS:000514673000032

Woll, S (2019) On visions and promises - ethical aspects of in vitro meat, EMERGING TOPICS IN LIFE SCIENCES, doi:10.1042/ETLS20190108 - WOS:000498896700015

Pali-Sch?ll, I; Binder, R; Moens, Y; Polesny, F; Mons?, S (2019) Edible insects ? defining knowledge gaps in biological and ethical considerations of entomophagy, CRITICAL REVIEWS IN FOOD SCIENCE AND NUTRITION, doi:10.1080/10408398.2018.1468731 - WOS:000489010500005

Michel, F; Siegrist, M (2019) How should importance of naturalness be measured? A comparison of different scales, APPETITE, doi:10.1016/j.appet.2019.05.019 - WOS:000473378800031

Grasso, AC; Hung, Y; Olthof, MR; Verbeke, W; Brouwer, IA (2019) Older Consumers' Readiness to Accept Alternative, More Sustainable Protein Sources in the European Union, NUTRIENTS, doi:10.3390/nu11081904 - WOS:000484506000225

Burton, RJF (2019) The potential impact of synthetic animal protein on livestock production: The new "war against agriculture"?, JOURNAL OF RURAL STUDIES, doi:10.1016/j.jrurstud.2019.03.002 - WOS:000470043600004

Alvaro, C (2019) Lab-Grown Meat and Veganism: A Virtue-Oriented Perspective, JOURNAL OF AGRICULTURAL & ENVIRONMENTAL ETHICS, doi:10.1007/s10806-019-09759-2 - WOS:000467100400008

Lo Sapio, L (2019) IN VITRO MEAT, A FLYWHEEL TO BUILD A NEW RELATIONSHIP BETWEEN SAPIENS AND NON-HUMAN ANIMALS, S&F-SCIENZAEFILOSOFIA IT, doi: - WOS:000504034700021

Hossain, MS (2019) CONSUMPTION OF STEM CELL MEAT: AN ISLAMIC PERSPECTIVE, IIUM LAW JOURNAL, doi:10.31436/iiumlj.v27i1.384 - WOS:000473305200009

Bohm, I; Ferrari, A; Woll, S (2018) Visions of In Vitro Meat among Experts and Stakeholders, NANOETHICS, doi:10.1007/s11569-018-0330-0 - WOS:000451025200004

Acevedo, CA; Orellana, N; Avarias, K; Ortiz, R; Benavente, D; Prieto, P (2018) Micropatterning Technology to Design an Edible Film for In Vitro Meat Production, FOOD AND BIOPROCESS TECHNOLOGY, doi:10.1007/s11947-018-2095-4 - WOS:000435413500001

Shriver, A; McConnachie, E (2018) Genetically Modifying Livestock for Improved Welfare: A Path Forward, JOURNAL OF AGRICULTURAL & ENVIRONMENTAL ETHICS, doi:10.1007/s10806-018-9719-6 - WOS:000431194400003

Lee, A (2018) Meat-ing Demand: Is In Vitro Meat a Pragmatic, Problematic, or Paradoxical Solution?, CANADIAN JOURNAL OF WOMEN AND THE LAW, doi:10.3138/cjwl.30.1.1 - WOS:000429822200001

[Anonymous] (2018) Location Germany continues to be attractive Consumer Preferences for vegan Foods, Insect Protein and in vitro Meat are low, FLEISCHWIRTSCHAFT, doi: - WOS:000444782400026

Woll, S; Bohm, I (2018) In Vitro Meat: A Solution to the Problems of Meat Production and the Meat Consumption?, ERNAHRUNGS UMSCHAU, doi:10.4455/eu.2018.003 - WOS:000425206000005

Enrione, J; Blaker, JJ; Brown, DI; Weinstein-Oppenheimer, CR; Pepczynska, M; Olguin, Y; Sanchez, E; Acevedo, CA (2017) Edible Scaffolds Based on Non-Mammalian Biopolymers for Myoblast Growth, MATERIALS, doi:10.3390/ma10121404 - WOS:000419207800064

Boler, DD; Woerner, DR (2017) What is meat? A perspective from the American Meat Science Association, ANIMAL FRONTIERS, doi:10.2527/af.2017.0436 - WOS:000457269700003

Henchion, M; Hayes, M; Mullen, AM; Fenelon, M; Tiwari, B (2017) Future Protein Supply and Demand: Strategies and Factors Influencing a Sustainable Equilibrium, FOODS, doi:10.3390/foods6070053 - WOS:000407336200008

Ferrari, A; Losch, A (2017) How Smart Grid Meets In Vitro Meat: on Visions as Socio-Epistemic Practices, NANOETHICS, doi:10.1007/s11569-017-0282-9 - WOS:000399179600008

Wilks, M; Phillips, CJC (2017) Attitudes to in vitro meat: A survey of potential consumers in the United States, PLOS ONE, doi:10.1371/journal.pone.0171904 - WOS:000394424500059

Bhat, ZF; Kumar, S; Bhat, HF (2017) In vitro meat: A future animal-free harvest, CRITICAL REVIEWS IN FOOD SCIENCE AND NUTRITION, doi:10.1080/10408398.2014.924899 - WOS:000390393300010

Jonsson, E (2016) Benevolent technotopias and hitherto unimaginable meats: Tracing the promises of in vitro meat, SOCIAL STUDIES OF SCIENCE, doi:10.1177/0306312716658561 - WOS:000386013900004

Milburn, J (2016) Chewing Over In Vitro Meat: Animal Ethics, Cannibalism and Social Progress, RES PUBLICA-A JOURNAL OF MORAL LEGAL AND POLITICAL PHILOSOPHY, doi:10.1007/s11158-016-9331-4 - WOS:000408726300001

Guermazi, M; Derbel, N; Kanoun, O (2016) Fuzzy logic diagnosis of the in-Vitro meat inspection based on Impedance Spectroscopy, PROCEEDINGS OF 2016 8TH INTERNATIONAL CONFERENCE ON MODELLING, IDENTIFICATION & CONTROL (ICMIC 2016), doi: - WOS:000392692800185

Singer, P (2016) IN VITRO MEAT, ETHICS IN THE REAL WORLD: 82 BRIEF ESSAYS ON THINGS THAT MATTER, doi: - WOS:000457328600017

Laestadius, LI; Caldwell, MA (2015) Is the future of meat palatable? Perceptions of in vitro meat as evidenced by online news comments, PUBLIC HEALTH NUTRITION, doi:10.1017/S1368980015000622 - WOS:000361067000019

Joachim, M (2015) A Century of Ecological Innovation, ARCHITECTURAL DESIGN, doi:10.1002/ad.1928 - WOS:000357810500011

Pandurangan, M; Kim, DH (2015) A novel approach for in vitro meat production, APPLIED MICROBIOLOGY AND BIOTECHNOLOGY, doi:10.1007/s00253-015-6671-5 - WOS:000356805600003

Chen, HG; Zhang, YHP (2015) New biorefineries and sustainable agriculture: Increased food, biofuels, and ecosystem security, RENEWABLE & SUSTAINABLE ENERGY REVIEWS, doi:10.1016/j.rser.2015.02.048 - WOS:000353755100011

Ubbink, J (2015) The In Vitro Meat Cookbook The Lab Grown Hamburger and 45 Other Recipes, SCIENCE, doi: - WOS:000351219600027

Hocquette, A; Lambert, C; Sinquin, C; Peterolff, L; Wagner, Z; Bonny, SPF; Lebert, A; Hocquette, JF (2015) Educated consumers don't believe artificial meat is the solution to the problems with the meat industry, JOURNAL OF INTEGRATIVE AGRICULTURE, doi:10.1016/S2095-3119(14)60886-8 - WOS:000349516100011

Galusky, W (2014) Technology as Responsibility: Failure, Food Animals, and Lab-grown Meat, JOURNAL OF AGRICULTURAL & ENVIRONMENTAL ETHICS, doi:10.1007/s10806-014-9508-9 - WOS:000345641600004

Catts, O; Zurr, I (2014) Growing for different ends, INTERNATIONAL JOURNAL OF BIOCHEMISTRY & CELL BIOLOGY, doi:10.1016/j.biocel.2014.09.025 - WOS:000347127400004

Tucker, CA (2014) The significance of sensory appeal for reduced meat consumption, APPETITE, doi:10.1016/j.appet.2014.06.022 - WOS:000340987000023

Schaefer, GO; Savulescu, J (2014) The Ethics of Producing InVitro Meat, JOURNAL OF APPLIED PHILOSOPHY, doi:10.1111/japp.12056 - WOS:000335402100006

Raimondi, S; Popovic, M; Amaretti, A; Di Gioia, D; Rossi, M (2014) Anti-Listeria Starters: In Vitro Selection and Production Plant Evaluation, JOURNAL OF FOOD PROTECTION, doi:10.4315/0362-028X.JFP-13-297 - WOS:000335432600021

Chiles, RM (2013) If they come, we will build it: in vitro meat and the discursive struggle over future agrofood expectations, AGRICULTURE AND HUMAN VALUES, doi:10.1007/s10460-013-9427-9 - WOS:000328052300003

Young, JF; Therkildsen, M; Ekstrand, B; Che, BN; Larsen, MK; Oksbjerg, N; Stagsted, J (2013) Novel aspects of health promoting compounds in meat, MEAT SCIENCE, doi:10.1016/j.meatsci.2013.04.036 - WOS:000323189100015

Chiles, RM (2013) Intertwined ambiguities: Meat, in vitro meat, and the ideological construction of the marketplace, JOURNAL OF CONSUMER BEHAVIOUR, doi:10.1002/cb.1447 - WOS:000327221000005

Stephens, N (2013) Growing Meat in Laboratories: The Promise, Ontology, and Ethical Boundary-Work of Using Muscle Cells to Make Food, CONFIGURATIONS, doi: - WOS:000327805900003

Cole, M; Morgan, K (2013) Engineering Freedom? A Critique of Biotechnological Routes to Animal Liberation, CONFIGURATIONS, doi: - WOS:000327805900005

Carruth, A (2013) Culturing Food: Bioart and In Vitro Meat, PARALLAX, doi:10.1080/13534645.2013.743296 - WOS:000314632600009

Driessen, C; Korthals, M (2012) Pig towers and in vitro meat: Disclosing moral worlds by design, SOCIAL STUDIES OF SCIENCE, doi:10.1177/0306312712457110 - WOS:000310957700001

Zurr, I; Catts, O (2012) Framing in-vitro meat as art, JOURNAL OF TISSUE ENGINEERING AND REGENERATIVE MEDICINE, doi: - WOS:000308313003201

Mironov, V; Genovese, NJ (2012) Enabling technologies for scalable production of in vitro meat, JOURNAL OF TISSUE ENGINEERING AND REGENERATIVE MEDICINE, doi: - WOS:000308313003205

Bax, ML; Aubry, L; Ferreira, C; Daudin, JD; Gatellier, P; Remond, D; Sante-Lhoutellier, V (2012) Cooking Temperature Is a Key Determinant of in Vitro Meat Protein Digestion Rate: Investigation of Underlying Mechanisms, JOURNAL OF AGRICULTURAL AND FOOD CHEMISTRY, doi:10.1021/jf205280y - WOS:000301407000022

Harvey, O (2012) Stem cell treatments in a global marketplace, COMMERCIALIZING THE STEM CELL SCIENCES, doi: - WOS:000324251900002

Pluhar, EB (2010) Meat and Morality: Alternatives to Factory Farming, JOURNAL OF AGRICULTURAL & ENVIRONMENTAL ETHICS, doi:10.1007/s10806-009-9226-x - WOS:000281727900004

Boonen, KJM; Langelaan, MLP; Polak, RB; van der Schaft, DWJ; Baaijens, FPT; Post, MJ (2010) Effects of a combined mechanical stimulation protocol: Value for skeletal muscle tissue engineering, JOURNAL OF BIOMECHANICS, doi:10.1016/j.jbiomech.2010.01.039 - WOS:000278652000012

Datar, I; Betti, M (2010) Possibilities for an in vitro meat production system, INNOVATIVE FOOD SCIENCE & EMERGING TECHNOLOGIES, doi:10.1016/j.ifset.2009.10.007 - WOS:000274600100002

Galusky, W (2010) Playing Chicken: Technologies of Domestication, Food, and Self, SCIENCE AS CULTURE, doi:10.1080/09505430903557874 - WOS:000290311500002

Parry, J (2009) Oryx and Crake and the New Nostalgia for Meat, SOCIETY & ANIMALS, doi:10.1163/156853009X445406 - WOS:000267724000004

Ren, B; Pulvers, E; Corbett, VN; Ghani, F; Liu, R; Anderson, K; Li, XK (2005) Are transverse strain and longitudinal strain determined by 2D strain method capable of quantifying myocardial muscle motion in layers with differing fiber orientation? Comparison with 2D tissue Doppler Imaging in an in vitro meat model, CIRCULATION, doi: - WOS:000232956403480

Tang, SZ; Kerry, JP; Sheehan, D; Buckley, DJ (2002) Antioxidative mechanisms of tea catechins in chicken meat systems, FOOD CHEMISTRY, doi:10.1016/S0308-8146(01)00248-5 - WOS:000172743400007

VANNETTEN, P; HUISINTVELD, J; MOSSEL, DAA (1994) AN IN-VITRO MEAT MODEL FOR THE IMMEDIATE BACTERICIDAL EFFECT OF LACTIC-ACID DECONTAMINATION ON MEAT SURFACES, JOURNAL OF APPLIED BACTERIOLOGY, doi: - WOS:A1994MU66400007

clean meat :

[Anonymous] (2020) Meat from the Petri Dish Clean Meat - caught between Origin, Motivation and Feasibility, FLEISCHWIRTSCHAFT, doi: - WOS:000510959900008

Tewfik, A; Babcock, EA; Appeldoorn, RS; Gibson, J (2019) Declining size of adults and juvenile harvest threatens sustainability of a tropical gastropod, Lobatus gigas, fishery, AQUATIC CONSERVATION-MARINE AND FRESHWATER ECOSYSTEMS, doi:10.1002/aqc.3147 - WOS:000494225300001

Thorrez, L; Vandenburgh, H (2019) Challenges in the quest for 'clean meat', NATURE BIOTECHNOLOGY, doi:10.1038/s41587-019-0043-0 - WOS:000460155900007

[Anonymous] (2019) German Meat Congress 2019 The Future begins now - between Livestock Strategy and Clean Meat, FLEISCHWIRTSCHAFT, doi: - WOS:000499765200059

Lynch, GS; Koopman, R (2019) Overcoming nature's paradox in skeletal muscle to optimise animal production, ANIMAL PRODUCTION SCIENCE, doi:10.1071/AN19361 - WOS:000489063400003

Windhorst, HW (2019) Meat and Fish from Cell Cultures Clean Meat as a Trend that has come to stay, FLEISCHWIRTSCHAFT, doi: - WOS:000461530700019

Bomgardner, M (2018) The to-do list for 'clean' meat, CHEMICAL & ENGINEERING NEWS, doi: - WOS:000448232500028

Voelker, R (2018) FDA Prods "Clean Meat" Discussion, JAMA-JOURNAL OF THE AMERICAN MEDICAL ASSOCIATION, doi:10.1001/jama.2018.9507 - WOS:000438900300007

Wird, F; Specht, L (2018) "We know it's possible" Liz Specht from the Good Food Institute in Los Angeles for the State of Sesearch in Clean Meat, FLEISCHWIRTSCHAFT, doi: - WOS:000444782400002

Windhorst, HW (2018) Clean Meat - The Beginning of a Revolution? Differences between Meat Substitutes and Meat from Cell Sultures are often unclear, FLEISCHWIRTSCHAFT, doi: - WOS:000434749700018

Lagally, C; Specht, L (2017) Opportunities for Translating Large-scale Cell Culture Technologies to the Production of Sustainable Clean Meat., IN VITRO CELLULAR & DEVELOPMENTAL BIOLOGY-ANIMAL, doi: - WOS:000402762600104

Makwana, PP; Nayak, JB; Brahmbhatt, MN; Chaudhary, JH (2015) Detection of Salmonella spp. from chevon, mutton and its environment in retail meat shops in Anand city (Gujarat), India, VETERINARY WORLD, doi:10.14202/vetworld.2015.388-392 - WOS:000217580900022

Nychas, GJE; Dourou, D; Skandamis, P; Koutsoumanis, K; Baranyi, J; Sofos, J (2009) Effect of microbial cell-free meat extract on the growth of spoilage bacteria, JOURNAL OF APPLIED MICROBIOLOGY, doi:10.1111/j.1365-2672.2009.04377.x - WOS:000271785400006

Kondaiah, N; Pragati, H (2005) Meat sector and its development, INDIAN JOURNAL OF ANIMAL SCIENCES, doi: - WOS:000234315800023

SPREULL, A (1962) PRACTICAL SLAUGHTERHOUSE CLEAN MEAT PRODUCTION, ROYAL SOCIETY OF HEALTH JOURNAL, doi:10.1177/146642406208200527 - WOS:A1962CDS6000040

[Anonymous] (1956) CLEAN MEAT, LANCET, doi: - WOS:A1956WL21900002

Egolf, A; Hartmann, C; Siegrist, M (2019) When Evolution Works Against the Future: Disgust's Contributions to the Acceptance of New Food Technologies, RISK ANALYSIS, doi:10.1111/risa.13279 - WOS:000474284900010

Liu, R; Wang, XD; Wang, XJ; Shi, YJ; Shi, C; Wang, W; Ma, CP (2019) A simple isothermal nucleic acid amplification method for the effective on site identification for adulteration of pork source in mutton, FOOD CONTROL, doi:10.1016/j.foodcont.2018.11.040 - WOS:000456754500039

Duconseille, A; Francois, O; Bruno, P; Celine, L; Marie-Agnes, P; Martine, H (2019) Measuring the effects of in vitro mastication on bolus granulometry of shredded meat: A proposal for a new methodological procedure, FOOD RESEARCH INTERNATIONAL, doi:10.1016/j.foodres.2018.10.014 - WOS:000458942900139

McGregor, A; Houston, D (2018) Cattle in the Anthropocene: Four propositions, TRANSACTIONS OF THE INSTITUTE OF BRITISH GEOGRAPHERS, doi:10.1111/tran.12193 - WOS:000425746100001

Geipel, J; Hadjichristidis, C; Klesse, AK (2018) Barriers to sustainable consumption attenuated by foreign language use, NATURE SUSTAINABILITY, doi:10.1038/s41893-017-0005-9 - WOS:000439103000014

Roos, E; Bajzelj, B; Smith, P; Patel, M; Little, D; Garnett, T (2017) Greedy or needy? Land use and climate impacts of food in 2050 under different livestock futures, GLOBAL ENVIRONMENTAL CHANGE-HUMAN AND POLICY DIMENSIONS, doi:10.1016/j.gloenvcha.2017.09.001 - WOS:000418392300001

Sodhi, N (2017) Artificial meat: a new taste sensation?, AUSTRALIAN VETERINARY JOURNAL, doi: - WOS:000411733300012

Roos, E; Bajzelj, B; Smith, P; Patel, M; Little, D; Garnett, T (2017) Protein futures for Western Europe: potential land use and climate impacts in 2050, REGIONAL ENVIRONMENTAL CHANGE, doi:10.1007/s10113-016-1013-4 - WOS:000394276200005

Hocquette, JF (2015) Is it possible to save the environment and satisify consumers with artificial meat?, JOURNAL OF INTEGRATIVE AGRICULTURE, doi:10.1016/S2095-3119(14)60961-8 - WOS:000349516100002

Orzechowski, A (2015) Artificial meat? Feasible approach based on the experience from cell culture studies, JOURNAL OF INTEGRATIVE AGRICULTURE, doi:10.1016/S2095-3119(14)60882-0 - WOS:000349516100004

Layman, DK (2014) Eating patterns, diet quality and energy balance A perspective about applications and future directions for the food industry, PHYSIOLOGY & BEHAVIOR, doi:10.1016/j.physbeh.2013.12.005 - WOS:000341475900019

Hocquette, JF; Minsant, P; Daudin, JD; Cassar-Malek, I; Remond, D; Doreau, M; Sans, P; Bauchart, D; Agabriel, J; Verbeke, W; Picard, B (2013) Will meat be produced in vitro in the future?, INRA PRODUCTIONS ANIMALES, doi: - WOS:000338001600005

McHugh, S (2010) Real Artificial: Tissue-cultured Meat, Genetically Modified Farm Animals, and Fictions, CONFIGURATIONS, doi:10.1353/con.2010.0006 - WOS:000290060500010

Arneth, W (2001) Chemistry of curing meat flavour, FLEISCHWIRTSCHAFT, doi: - WOS:000167599600005

Daschner, F; Kent, M; Knochel, R; Berger, UK (2000) Multiparameter microwave sensors for determining composition or condition of substances, 2000 IEEE MTT-S INTERNATIONAL MICROWAVE SYMPOSIUM DIGEST, VOLS 1-3, doi: - WOS:000166811000367

Bengtsson, GB; Skorbakk, OI (1997) Stability of Prussian blue bound to anion-exchange resin beads for radiocaesium reduction in foodstuffs, FOOD CHEMISTRY, doi:10.1016/S0308-8146(96)00000-3 - WOS:A1997WW48200014

Sutherland, WJ; Fleishman, E; Clout, M; Gibbons, DW; Lickorish, F; Peck, LS; Pretty, J; Spalding, M; Ockendon, N (2019) Ten Years On: A Review of the First Global Conservation Horizon Scan, TRENDS IN ECOLOGY & EVOLUTION, doi:10.1016/j.tree.2018.12.003 - WOS:000456952500006

Lo Sapio, L (2019) THE FOURTH INDUSTRIAL REVOLUTION. PHILOSOPHICAL FRAMEWORK AND ETHICAL MODELS, S&F-SCIENZAEFILOSOFIA IT, doi: - WOS:000504034700006

Danaee, S; Ofoghi, H; Heydarian, SM; Farazmand, A (2018) Optimization of nitrogen and phosphorus removal from meat processing wastewaters using microalgal biofilms, ENVIRONMENTAL HEALTH ENGINEERING AND MANAGEMENT JOURNAL, doi:10.15171/EHEM.2018.12 - WOS:000452678800004

Jones, S (2017) Synthetic meat offers surprising possibilities, NEW SCIENTIST, doi: - WOS:000393736500043

Sharma, SK; Bansal, S; Mangal, M; Dixit, AK; Gupta, RK; Mangal, AK (2016) Utilization of Food Processing By-products as Dietary, Functional, and Novel Fiber: A Review, CRITICAL REVIEWS IN FOOD SCIENCE AND NUTRITION, doi:10.1080/10408398.2013.794327 - WOS:000379589400006

Marcu, A; Gaspar, R; Rutsaert, P; Seibt, B; Fletcher, D; Verbeke, W; Barnett, J (2015) Analogies, metaphors, and wondering about the future: Lay sense-making around synthetic meat, PUBLIC UNDERSTANDING OF SCIENCE, doi:10.1177/0963662514521106 - WOS:000356310100003

Moon, JH; Choi, IW; Park, YK; Kim, Y (2011) Development of Natural Meat-like Flavor Based on Mail lard Reaction Products, KOREAN JOURNAL FOR FOOD SCIENCE OF ANIMAL RESOURCES, doi:10.5851/kosfa.2011.31.1.129 - WOS:000288353000019

Noriega, E; Laca, A; Diaz, M (2009) Listeria growth under diffusional limitations in synthetic meats, INTERNATIONAL JOURNAL OF FOOD SCIENCE AND TECHNOLOGY, doi:10.1111/j.1365-2621.2008.01885.x - WOS:000264185700010

Yap, KH; Kadim, IT; King, RD; Moughan, PJ (1997) An ileal amino acid digestibility assay for the growing meat chicken-effect of feeding method and digesta collection procedures, ASIAN-AUSTRALASIAN JOURNAL OF ANIMAL SCIENCES, doi:10.5713/ajas.1997.671 - WOS:000071183300020

STEPHENSON, T; LESTER, JN (1986) EVALUATION OF STARTUP AND OPERATION OF 4 ANAEROBIC PROCESSES TREATING A SYNTHETIC MEAT WASTE, BIOTECHNOLOGY AND BIOENGINEERING, doi:10.1002/bit.260280310 - WOS:A1986A241900009

RUDD, T; HICKS, SJ; LESTER, JN (1985) COMPARISON OF THE TREATMENT OF A SYNTHETIC MEAT WASTE BY MESOPHILIC AND THERMOPHILIC ANAEROBIC FLUIDIZED-BED REACTORS, ENVIRONMENTAL TECHNOLOGY LETTERS, doi:10.1080/09593338509384338 - WOS:A1985AJP1500004

HSIEH, YPC; PEARSON, AM; SWEELEY, CC; MARTIN, FE (1980) USE OF SPECTRAL SEARCH FOR IDENTIFICATION OF THE VOLATILES IN A SYNTHETIC MEAT FLAVOR SYSTEM, JOURNAL OF FOOD SCIENCE, doi:10.1111/j.1365-2621.1980.tb07523.x - WOS:A1980JX87400082

HSIEH, YPC; PEARSON, AM; MAGEE, WT (1980) DEVELOPMENT OF A SYNTHETIC MEAT FLAVOR MIXTURE BY USING SURFACE RESPONSE METHODOLOGY, JOURNAL OF FOOD SCIENCE, doi:10.1111/j.1365-2621.1980.tb06502.x - WOS:A1980KH30300006

MCCARNEY, LJ (1975) COMMUNICATION PROBLEMS IN MARKETING OF SYNTHETIC MEATS, EUROPEAN JOURNAL OF MARKETING, doi:10.1108/EUM0000000005067 - WOS:A1975BA78800002

SCHRODER, DJ; BUSTA, FF (1973) EFFECTS OF SYNTHETIC MEAT COMPONENTS ON GROWTH OF CLOSTRIDIUM-PERFRINGENS, JOURNAL OF MILK AND FOOD TECHNOLOGY, doi:10.4315/0022-2747-36.4.189 - WOS:A1973P460300001

Swartz, E (2019) Meeting the Needs of the Cell-Based Meat Industry, CHEMICAL ENGINEERING PROGRESS, doi: - WOS:000491214100016

Valente, JDS; Fiedler, RA; Heidemann, MS; Molento, CFM (2019) First glimpse on attitudes of highly educated consumers towards cell-based meat and related issues in Brazil, PLOS ONE, doi:10.1371/journal.pone.0221129 - WOS:000485062200011

Legendre, N (2019) Automation in Cell-based Meat Production., IN VITRO CELLULAR & DEVELOPMENTAL BIOLOGY-ANIMAL, doi: - WOS:000467573200024

Specht, L (2019) Translating Large-scale Commercial Cell Culture Technologies to the Production of Cell-based Meat., IN VITRO CELLULAR & DEVELOPMENTAL BIOLOGY-ANIMAL, doi: - WOS:000467573200070

Sancar, F (2019) Agreement to Regulate Cell-Based Meat Products, JAMA-JOURNAL OF THE AMERICAN MEDICAL ASSOCIATION, doi:10.1001/jama.2019.3831 - WOS:000464765900032

Johnson, WG (2019) Conflict Over Cell-Based Meat: Who Should Coordinate Agencies in US Biotechnology Regulation?, FOOD AND DRUG LAW JOURNAL, doi: - WOS:000504365600006

Voelker, R (2018) Cardiologist Trades Stem Cells for Cell-Based Meat, JAMA-JOURNAL OF THE AMERICAN MEDICAL ASSOCIATION, doi:10.1001/jama.2018.11895 - WOS:000446143400001

Dolgin, E (2019) Lab-grown meat gets rare funding boost, NATURE, doi:10.1038/d41586-019-00373-w - WOS:000458503900013

Circus, VE; Robison, R (2019) Exploring perceptions of sustainable proteins and meat attachment, BRITISH FOOD JOURNAL, doi:10.1108/BFJ-01-2018-0025 - WOS:000468888100022

Mayhall, TA (2019) The Meat of the Matter: Regulating a Laboratory-Grown Alternative, FOOD AND DRUG LAW JOURNAL, doi: - WOS:000462422900006

Smetana, S; Mathys, A; Knoch, A; Heinz, V (2015) Meat alternatives: life cycle assessment of most known meat substitutes, INTERNATIONAL JOURNAL OF LIFE CYCLE ASSESSMENT, doi:10.1007/s11367-015-0931-6 - WOS:000359739200005

Bomgardner, M (2019) Fake meat firms gain steam, CHEMICAL & ENGINEERING NEWS, doi: - WOS:000486563500023

Clark, LF; Bogdan, AM (2019) The Role of Plant-Based Foods in Canadian Diets: A Survey Examining Food Choices, Motivations and Dietary Identity, JOURNAL OF FOOD PRODUCTS MARKETING, doi:10.1080/10454446.2019.1566806 - WOS:000466789400001

DeFrancesco, L (2019) Not 'fake' meat but 'clean', NATURE BIOTECHNOLOGY, doi:10.1038/nbt0119-9b - WOS:000473593000002

Henderson, S (2018) Fake meat and artisanal food, farming and tonics, NEW SCIENTIST, doi: - WOS:000433378000031

Grimstead, D (2018) Fake meat and artisanal food, farming and tonics, NEW SCIENTIST, doi: - WOS:000433378000032

Lee, JN; Jiang, MF; Wen, YL; Li, SL; Yuan, GR (2018) Multiplex Assay for Identifying Animal Species Found in the Tibetan Area Using the Mitochondrial 12S rRNA Gene, ANIMAL BIOTECHNOLOGY, doi:10.1080/10495398.2017.1350690 - WOS:000419944000010

Fischer, B; Ozturk, B (2017) Facsimiles of Flesh, JOURNAL OF APPLIED PHILOSOPHY, doi:10.1111/japp.12223 - WOS:000407248900003

Fellet, M (2015) A Fresh Take on Fake Meat, ACS CENTRAL SCIENCE, doi:10.1021/acscentsci.5b00307 - WOS:000365969500003

[Anonymous] (2015) The Problem with Fake Meat, TECHNOLOGY REVIEW, doi: - WOS:000369555100005

Dai, C; Jiang, M (2013) Fake meat scandals add to Chinese food fears, BMJ-BRITISH MEDICAL JOURNAL, doi:10.1136/bmj.f3385 - WOS:000319947500012

Huang, SH (2012) The Authenticity of Fake Meats, ISLE-INTERDISCIPLINARY STUDIES IN LITERATURE AND ENVIRONMENT, doi:10.1093/isle/iss108 - WOS:000315642400008

Pelletier, MG; Holt, GA; Wanjura, JD; Greetham, L; McIntyre, G; Bayer, E; Kaplan-Bie, J (2019) Acoustic evaluation of mycological biopolymer, an all-natural closed cell foam alternative, INDUSTRIAL CROPS AND PRODUCTS, doi:10.1016/j.indcrop.2019.111533 - WOS:000484646900081

Segovia-Siapco, G; Sabate, J (2019) Health and sustainability outcomes of vegetarian dietary patterns: a revisit of the EPIC-Oxford and the Adventist Health Study-2 cohorts, EUROPEAN JOURNAL OF CLINICAL NUTRITION, doi:10.1038/s41430-018-0310-z - WOS:000489111000008

Ullah, H; Santos, HA; Khan, T (2016) Applications of bacterial cellulose in food, cosmetics and drug delivery, CELLULOSE, doi:10.1007/s10570-016-0986-y - WOS:000380089300003

Spencer, RJ; Russell, JM; Barker, ME (2014) Temporality in British young women's magazines: food, cooking and weight loss, PUBLIC HEALTH NUTRITION, doi:10.1017/S1368980013002620 - WOS:000344544100027

De Keyzer, W; Van Caneghem, S; Heath, ALM; Vanaelst, B; Verschraegen, M; De Henauw, S; Huybrechts, I (2012) Nutritional quality and acceptability of a weekly vegetarian lunch in primary-school canteens in Ghent, Belgium: 'Thursday Veggie Day', PUBLIC HEALTH NUTRITION, doi:10.1017/S1368980012000870 - WOS:000311285500022

Havlik, J; Plachy, V; Fernandez, J; Rada, V (2010) Dietary purines in vegetarian meat analogues, JOURNAL OF THE SCIENCE OF FOOD AND AGRICULTURE, doi:10.1002/jsfa.4089 - WOS:000283818200002

Dietrich, R; Usleber, E; Martlbauer, E; Gareis, M (1999) Detection of the nephrotoxic mycotoxin citrinin in foods and food colorants derived from Monascus spp, ARCHIV FUR LEBENSMITTELHYGIENE, doi: - WOS:000079081500004

Bomgardner, M (2018) Animal-free meat, dairy firms plan scale-up, CHEMICAL & ENGINEERING NEWS, doi: - WOS:000451360400012

Fowler, KW (2018) Terms for animal-free meat, CHEMICAL & ENGINEERING NEWS, doi: - WOS:000451106600004

Bomgardner, M (2018) Animal-free meat, dairy firms raise funds, CHEMICAL & ENGINEERING NEWS, doi: - WOS:000424068800020

Ceurstemont, S (2017) Make your own meat Can we grow animal-free meat locally, NEW SCIENTIST, doi: - WOS:000391912200023

[Anonymous] (2015) Sustainable Proteins: From Crickets to Test-Tube Meat, FOOD TECHNOLOGY, doi: - WOS:000351336500007

[Anonymous] (2013) Serving up test-tube meat, FOOD TECHNOLOGY, doi: - WOS:000326663800011

[Anonymous] (2012) TEST TUBE MEAT, FOOD AUSTRALIA, doi: - WOS:000303701000005

Fox, JL (2009) Test tube meat on the menu?, NATURE BIOTECHNOLOGY, doi:10.1038/nbt1009-873 - WOS:000271472500003

Borning, J; Tiberius, V (2017) By 2027, a Research Focus lies on Mass Production of cultivated Meat Market- and Technology Perspectives for in-vitro-Meat: A Delphi-Study for the Year 2027, FLEISCHWIRTSCHAFT, doi: - WOS:000411554400024

Mattice, KD; Marangoni, AG (2020) Comparing methods to produce fibrous material from zein, FOOD RESEARCH INTERNATIONAL, doi:10.1016/j.foodres.2019.108804 - WOS:000510954200033

Weber, H (2018) Meat Extensions - Products containing aanimal and vegetable Components Products with reduced Meat Content as an Alternative to vegetarian and vegan Meat Analogues, FLEISCHWIRTSCHAFT, doi: - WOS:000434104600033

Campbell, L (2017) Caribbean Vegan: Meat Free, Egg-Free, Dairy-Free, Authentic Island Cuisine for Every Occasion., LIBRARY JOURNAL, doi: - WOS:000394201700208

Metcalf, J (2013) Meet Shmeat: Food System Ethics, Biotechnology and Re-Worlding Technoscience, PARALLAX, doi:10.1080/13534645.2013.743294 - WOS:000314632600008

Bierbaum, R; Leonard, SA; Rejeski, D; Whaley, C; Barra, RO; Libre, C (2020) Novel entities and technologies: Environmental benefits and risks, ENVIRONMENTAL SCIENCE & POLICY, doi:10.1016/j.envsci.2019.11.002 - WOS:000513988400012

Bryant, CJ (2019) We Can't Keep Meating Like This: Attitudes towards Vegetarian and Vegan Diets in the United Kingdom, SUSTAINABILITY, doi:10.3390/su11236844 - WOS:000508186400316

Broad, GM (2019) Plant-based and cell-based animal product alternatives: An assessment and agenda for food tech justice, GEOFORUM, doi:10.1016/j.geoforum.2019.06.014 - WOS:000503090800023

Giraldo, M; Buodo, G; Sarlo, M (2019) Food processing and emotion regulation in vegetarians and omnivores: An event-related potential investigation, APPETITE, doi:10.1016/j.appet.2019.104334 - WOS:000477690300033

Herbert, R; Mishra, S; Lim, HR; Yoo, H; Yeo, WH (2019) Fully Printed, Wireless, Stretchable Implantable Biosystem toward Batteryless, Real-Time Monitoring of Cerebral Aneurysm Hemodynamics, ADVANCED SCIENCE, doi:10.1002/advs.201901034 - WOS:000479064100001

Rehman, A; Zhang, DY; Chandio, AA (2019) Contribution of Beef, Mutton, and Poultry Meat Production to the Agricultural Gross Domestic Product of Pakistan Using an Autoregressive Distributed Lag Bounds Testing Approach, SAGE OPEN, doi:10.1177/2158244019877196 - WOS:000487132700001

Meyfroidt, P; Abeygunawardane, D; Ramankutty, N; Thomson, A; Zeleke, G (2019) Interactions between land systems and food systems, CURRENT OPINION IN ENVIRONMENTAL SUSTAINABILITY, doi:10.1016/j.cosust.2019.04.010 - WOS:000480419900010

Liu, SL; Gasteratos, K (2019) Assessing cell-based animal proteins, SCIENCE, doi:10.1126/science.aau3905 - WOS:000459387100032

Wurgaft, BA (2019) Meat Planet: Artificial Flesh and the Future of Food, MEAT PLANET: ARTIFICIAL FLESH AND THE FUTURE OF FOOD, doi: - WOS:000517449700019

Windhorst, HW (2019) Who comes too late, is left with nothing The Dynamics in the Development of alternative Meat Products are underestimated, FLEISCHWIRTSCHAFT, doi: - WOS:000499765200014

Lim, JTS (2019) New Innovation on Vegan Food, ANNALS OF NUTRITION AND METABOLISM, doi: - WOS:000480262700120

Wells, M (2019) Development of Environmentally Sustainable Materials, ECOLOGICAL WISDOM INSPIRED RESTORATION ENGINEERING, doi:10.1007/978-981-13-0149-0_1 - WOS:000481828200003

Wagner, J; Joldic, A (2018) Hydrocolloids as Base for Squid Rings Development of an innovative Process for the Production of vegan Fish Alternatives, FLEISCHWIRTSCHAFT, doi: - WOS:000454972200013

Hamm, MW (2018) Sustainable protein provisioning, NATURE SUSTAINABILITY, doi:10.1038/s41893-018-0196-8 - WOS:000453337000008

Bomgardner, M (2018) FDA, USDA to oversee cell -based meat, CHEMICAL & ENGINEERING NEWS, doi: - WOS:000451360400022

Dekkers, BL; Boom, RM; van der Goot, AJ (2018) Structuring processes for meat analogues, TRENDS IN FOOD SCIENCE & TECHNOLOGY, doi:10.1016/j.tifs.2018.08.011 - WOS:000449126900003

D'Odorico, P; Davis, KF; Rosa, L; Carr, JA; Chiarelli, D; Dell'Angelo, J; Gephart, J; MacDonald, GK; Seekell, DA; Suweis, S; Rulli, MC (2018) The Global Food-Energy-Water Nexus, REVIEWS OF GEOPHYSICS, doi:10.1029/2017RG000591 - WOS:000447805200001

Radice, A; Iorno, MLC; Meucci, E; Fassio, F; Manfredi, M; Macchia, D (2018) Biologic prosthesis allergic sensitization: What do we (not) know?, ALLERGY, doi: - WOS:000441690403058

van Vuuren, DP; Stehfest, E; Gernaat, DEHJ; van den Berg, M; Bijl, DL; de Boer, HS; Daioglou, V; Doelman, JC; Edelenbosch, OY; Harmsen, M; Hof, AF; van Sluisveld, MAE (2018) Alternative pathways to the 1.5 degrees C target reduce the need for negative emission technologies, NATURE CLIMATE CHANGE, doi:10.1038/s41558-018-0119-8 - WOS:000431139900021

Croney, C; Muir, W; Ni, JQ; Widmar, NO; Varner, G (2018) An Overview of Engineering Approaches to Improving Agricultural Animal Welfare, JOURNAL OF AGRICULTURAL & ENVIRONMENTAL ETHICS, doi:10.1007/s10806-018-9716-9 - WOS:000431194400002

Milburn, J (2018) Death-Free Dairy? The Ethics of Clean Milk, JOURNAL OF AGRICULTURAL & ENVIRONMENTAL ETHICS, doi:10.1007/s10806-018-9723-x - WOS:000431194400008

Qi, J; Li, X; Zhang, WW; Wang, HH; Zhou, GH; Xu, XL (2018) Influence of stewing time on the texture, ultrastructure and in vitro digestibility of meat from the yellow-feathered chicken breed, ANIMAL SCIENCE JOURNAL, doi:10.1111/asj.12929 - WOS:000424094900023

Specht, L (2018) Is the Future of Meat Animal-Free?, FOOD TECHNOLOGY, doi: - WOS:000442116100009

Olivero, R; Florez, A; Aguas, Y (2018) EVALUATION OF ACTIVE COMPONENTS OF CALATHEA LUTEA AS A COATING IN FOOD PRODUCTS, ANNALS OF NUTRITION AND METABOLISM, doi: - WOS:000437460400080

Cartin-Rojas, A (2017) Food fraud and adulteration: a challenge for the foresight of Veterinary Services, REVUE SCIENTIFIQUE ET TECHNIQUE-OFFICE INTERNATIONAL DES EPIZOOTIES, doi:10.20506/rst.36.3.2733 - WOS:000426414100025

van Huis, A; Oonincx, DGAB (2017) The environmental sustainability of insects as food and feed. A review, AGRONOMY FOR SUSTAINABLE DEVELOPMENT, doi:10.1007/s13593-017-0452-8 - WOS:000411086200007

Swick, B (2017) Foreword to "Recent Advances in Animal Nutrition in Australia, 2017', ANIMAL PRODUCTION SCIENCE, doi:10.1071/ANv57n11_FO - WOS:000412097600016

Cooper, MH (2017) Open Up and Say "Baa": Examining the Stomachs of Ruminant Livestock and the Real Subsumption of Nature, SOCIETY & NATURAL RESOURCES, doi:10.1080/08941920.2017.1295494 - WOS:000402012200003

Sijtsema, SJ; Onwezen, MC; Reinders, MJ; Dagevos, H; Partanen, A; Meeusen, M (2016) Consumer perception of bio-based products-An exploratory study in 5 European countries, NJAS-WAGENINGEN JOURNAL OF LIFE SCIENCES, doi:10.1016/j.njas.2016.03.007 - WOS:000377229200008

Comans, C; Langen, M; Horn, D (2016) LMIV Sausage or Non-Sausage, that is the Question, FLEISCHWIRTSCHAFT, doi: - WOS:000377848400015

Goldman, TR (2015) From Lab Bench To Kitchen Table, HEALTH AFFAIRS, doi:10.1377/hlthaff.2015.1127 - WOS:000366724000003

Mattick, CS; Wetmore, JM; Allenby, BR (2015) An Anticipatory Social Assessment of Factory-Grown Meat, IEEE TECHNOLOGY AND SOCIETY MAGAZINE, doi:10.1109/MTS.2015.2395967 - WOS:000351739300010

Loveridge, A; Cade, JE; Burley, VJ (2015) Meat eating and risk of arthritis in the UK Women's Cohort Study, PROCEEDINGS OF THE NUTRITION SOCIETY, doi:10.1017/S0029665115003377 - WOS:000362835100014

D'Odorico, P; Carr, JA; Laio, F; Ridolfi, L; Vandoni, S (2014) Feeding humanity through global food trade, EARTHS FUTURE, doi:10.1002/2014EF000250 - WOS:000358135000003

Griggs, AM; Agim, ZS; Mishra, VR; Tambe, MA; Director-Myska, AE; Turteltaub, KW; McCabe, GP; Rochet, JC; Cannon, JR (2014) 2-Amino-1-methyl-6-phenylimidazo[4,5-b]pyridine (PhIP) Is Selectively Toxic to Primary Dopaminergic Neurons In Vitro, TOXICOLOGICAL SCIENCES, doi:10.1093/toxsci/kfu060 - WOS:000339722100016

Mattick, C; Allenby, B (2013) The Future of Meat, ISSUES IN SCIENCE AND TECHNOLOGY, doi: - WOS:000329883100019

Bekhit, AEA; Hopkins, DL; Fahri, FT; Ponnampalam, EN (2013) Oxidative Processes in Muscle Systems and Fresh Meat: Sources, Markers, and Remedies, COMPREHENSIVE REVIEWS IN FOOD SCIENCE AND FOOD SAFETY, doi:10.1111/1541-4337.12027 - WOS:000324096800007

Sharma, S; Yoshida, S; Braff-Guajardo, E; Ritchie, LD (2013) Food Choices for 2-5 Year Old Children in California Childcare: Comparing 2008 and 2012, FASEB JOURNAL, doi: - WOS:000319883505194

Girish, PS; Reddy, BP; Ramakrishna, C; Reddy, YR; Chava, S; Kondaiah, N (2012) Effect of nutrient supplementation on growth performance and carcass characteristics of Nellore ram lambs - An on-farm evaluation study, INDIAN JOURNAL OF ANIMAL SCIENCES, doi: - WOS:000209113400032

Aksoy, A; Guven, A; Gulmez, M (2011) Decontamination and Shelf-life Potential of Some Herbal Infussions and Hydrodistillates on Chicken Meat, KAFKAS UNIVERSITESI VETERINER FAKULTESI DERGISI, doi: - WOS:000290955200023

Hwang, SC; Yao, C; Kuo, IY; Tsai, WC; Chang, H (2011) Tissue Necrosis Monitoring for HIFU Ablation with T1 Contrast MRI Imaging, 10TH INTERNATIONAL SYMPOSIUM ON THERAPEUTIC ULTRASOUND (ISTU 2010), doi:10.1063/1.3607898 - WOS:000295944900024

Jones, N (2010) A taste of things to come?, NATURE, doi:10.1038/468752a - WOS:000285093700015

Holker, F; Wolter, C; Perkin, EK; Tockner, K (2010) Light pollution as a biodiversity threat, TRENDS IN ECOLOGY & EVOLUTION, doi:10.1016/j.tree.2010.09.007 - WOS:000284795800001

Sutherland, WJ; Clout, M; Cote, IM; Daszak, P; Depledge, MH; Fellman, L; Fleishman, E; Garthwaite, R; Gibbons, DW; De Lurio, J; Impey, AJ; Lickorish, F; Lindenmayer, D; Madgwick, J; Margerison, C; Maynard, T; Peck, LS; Pretty, J; Prior, S; Redford, KH; Scharlemann, JPW; Spalding, M; Watkinson, AR (2010) A horizon scan of global conservation issues for 2010, TRENDS IN ECOLOGY & EVOLUTION, doi:10.1016/j.tree.2009.10.003 - WOS:000274074500001

Sen, CT (2009) Indian meals, MEALS IN SCIENCE AND PRACTICE: INTERDISCIPLINARY RESEARCH AND BUSINESS APPLICATIONS, doi:10.1533/9781845695712.6.394 - WOS:000276522300020

von Castel-Roberts, K; Kauwell, GPA; Maneval, D; Young, LJ; Nexo, E; Bailey, LB (2008) Vitamin B12 intake above current recommendations may be needed to ensure optimal vitamin B12 status in sub-groups of healthy adults, FASEB JOURNAL, doi: - WOS:000208467805240

Kuhnle, GGC; Bingham, SA (2007) Dietary meat, endogenous nitrosation and colorectal cancer, BIOCHEMICAL SOCIETY TRANSACTIONS, doi:10.1042/BST0351355 - WOS:000251279800122

Kelly, L (2007) Meat, in vitro?, SCIENTIST, doi: - WOS:000250495700010

Pincock, S (2007) Meat, in vitro?, SCIENTIST, doi: - WOS:000248988800010

Pincock, S (2007) Isle(t) of the pigs, SCIENTIST, doi: - WOS:000246893800014

Campo, MM; Nute, GR; Wood, JD; Elmore, SJ; Mottram, DS; Enser, M (2003) Modelling the effect of fatty acids in odour development of cooked meat in vitro: part I - sensory perception, MEAT SCIENCE, doi:10.1016/S0309-1740(02)00095-5 - WOS:000179484100013

Hua, NW; Stoohs, RA; Facchini, FS (2001) Low iron status and enhanced insulin sensitivity in lacto-ovo vegetarians, BRITISH JOURNAL OF NUTRITION, doi:10.1079/BJN2001421 - WOS:000171440900012

Iriarte, J; Castane, C (2001) Artificial rearing of Dicyphus tamaninii (Heteroptera : Miridae) on a meat-based diet, BIOLOGICAL CONTROL, doi:10.1006/bcon.2001.0951 - WOS:000170820200013

Borja, R; Banks, CJ; Wang, ZJ; Mancha, A (1998) Anaerobic digestion of slaughterhouse wastewater using a combination sludge blanket and filter arrangement in a single reactor, BIORESOURCE TECHNOLOGY, doi:10.1016/S0960-8524(98)00004-2 - WOS:000074638300017

Hugas, M (1998) Bacteriocinogenic lactic acid bacteria for the biopreservation of meat and meat products, MEAT SCIENCE, doi:10.1016/S0309-1740(98)90044-4 - WOS:000075885100011

Mann, JI; Appleby, PN; Key, TJ; Thorogood, M (1997) Dietary determinants of ischaemic heart disease in health conscious individuals, HEART, doi:10.1136/hrt.78.5.450 - WOS:A1997YG24300007

Finzi, A; Margarit, R; Macchioni, P (1997) Rabbit germplasm utilisation to produce a synthetic breed fit to Mediterranean climates, INTERNATIONAL SYMPOSIUM ON MEDITERRANEAN ANIMAL GERMPLASM AND FUTURE HUMAN CHALLENGES: A JOINT EAAP - FAO - CIHEAM INTERNATIONAL SYMPOSIUM, doi: - WOS:A1997BJ31G00029

BORJA, R; BANKS, CJ; WANG, ZJ (1995) EFFECT OF ORGANIC LOADING RATE ON ANAEROBIC TREATMENT OF SLAUGHTERHOUSE WASTE-WATER IN A FLUIDIZED-BED REACTOR, BIORESOURCE TECHNOLOGY, doi:10.1016/0960-8524(95)00017-9 - WOS:A1995RJ33300008

BORJA, R; BANKS, CJ; WANG, ZJ (1994) STABILITY AND PERFORMANCE OF AN ANAEROBIC DOWNFLOW FILTER TREATING SLAUGHTERHOUSE WASTE-WATER UNDER TRANSIENT CHANGES IN-PROCESS PARAMETERS, BIOTECHNOLOGY AND APPLIED BIOCHEMISTRY, doi: - WOS:A1994PU68000006

ANNACHHATRE, AP; BHAMIDIMARRI, SMR (1992) MICROBIAL ATTACHMENT AND GROWTH IN FIXED-FILM REACTORS - PROCESS STARTUP CONSIDERATIONS, BIOTECHNOLOGY ADVANCES, doi:10.1016/0734-9750(92)91352-F - WOS:A1992HP37700002

CHUDASAMA, Y; HAMILTONMILLER, JMT; MAPLE, PAC (1991) BACTERIOLOGICAL SAFETY OF COOK-CHILL FOOD AT THE ROYAL-FREE-HOSPITAL, WITH PARTICULAR REFERENCE TO LISTERIA, JOURNAL OF HOSPITAL INFECTION, doi:10.1016/0195-6701(91)90239-5 - WOS:A1991GY83900002

LOPRIENO, N; BONCRISTIANI, G; LOPRIENO, G (1991) AN EXPERIMENTAL APPROACH TO IDENTIFYING THE GENOTOXIC RISK FROM COOKED MEAT MUTAGENS, FOOD AND CHEMICAL TOXICOLOGY, doi:10.1016/0278-6915(91)90077-K - WOS:A1991GD65900003

VAGHEFI, SB; SAVILLE, W (1991) STUDY OF DIET AND LIFE-STYLE ON LOSS OF BONE CALCIUM IN PRE AND POSTMENOPAUSAL WOMEN, FASEB JOURNAL, doi: - WOS:A1991FC20701186

HICKEY, RF; WU, WM; VEIGA, MC; JONES, R (1991) START-UP, OPERATION, MONITORING AND CONTROL OF HIGH-RATE ANAEROBIC TREATMENT SYSTEMS, WATER SCIENCE AND TECHNOLOGY, doi: - WOS:A1991GG35900014

MILLS, PK; ANNEGERS, JF; PHILLIPS, RL (1988) ANIMAL PRODUCT CONSUMPTION AND SUBSEQUENT FATAL BREAST-CANCER RISK AMONG 7TH-DAY ADVENTISTS, AMERICAN JOURNAL OF EPIDEMIOLOGY, doi:10.1093/oxfordjournals.aje.a114821 - WOS:A1988M162600002

NITZSCHE, G; LIETZAU, H; HEINECKE, K (1988) BREEDING FOR A NEW MEAT-TYPE SIRE POPULATION - METHODS AND RESULTS, ARCHIV FUR TIERZUCHT-ARCHIVES OF ANIMAL BREEDING, doi: - WOS:A1988Q545900005

FEHRS, LJ; FLANAGAN, K; KLINE, S; FACKLAM, RR; QUACKENBUSH, K; FOSTER, LR (1987) GROUP-A BETA-HEMOLYTIC STREPTOCOCCAL SKIN INFECTIONS IN A UNITED-STATES MEAT-PACKING PLANT, JAMA-JOURNAL OF THE AMERICAN MEDICAL ASSOCIATION, doi:10.1001/jama.258.21.3131 - WOS:A1987L039900023

WAGNER, H (1987) AROMA-DEVELOPING SUBSTANCES IN MEAT, FLEISCHWIRTSCHAFT, doi: - WOS:A1987H485700008

GORDER, DD; DOLECEK, TA; COLEMAN, GG; TILLOTSON, JL; BROWN, HB; LENZLITZOW, K; BARTSCH, GE; GRANDITS, G (1986) DIETARY-INTAKE IN THE MULTIPLE RISK FACTOR INTERVENTION TRIAL (MRFIT) - NUTRIENT AND FOOD GROUP CHANGES OVER 6 YEARS, JOURNAL OF THE AMERICAN DIETETIC ASSOCIATION, doi: - WOS:A1986C659500003

MCNEILL, DA; ALI, PS; SONG, YS (1985) MINERAL ANALYSES OF VEGETARIAN, HEALTH, AND CONVENTIONAL FOODS - MAGNESIUM, ZINC, COPPER, AND MANGANESE CONTENT, JOURNAL OF THE AMERICAN DIETETIC ASSOCIATION, doi: - WOS:A1985AGS4100004

BARNETT, D; HOWDEN, MEH (1984) A ROCKET IMMUNOELECTROPHORETIC METHOD FOR THE DETECTION OF HEAT-TREATED PEANUT PROTEIN, FOOD TECHNOLOGY IN AUSTRALIA, doi: - WOS:A1984TR28200005

DANEHY, JP; WOLNAK, B (1983) MAILLARD TECHNOLOGY - MANUFACTURING APPLICATIONS IN FOOD-PRODUCTS, ACS SYMPOSIUM SERIES, doi: - WOS:A1983QM82800014

SIPPACH, G; VIERLING, P; DIETSCH, K (1982) HIGH-TEMPERATURE MATURATION OF DURABLE SALT MEAT UNDER MICROBIOLOGICAL CONTROL, FLEISCH, doi: - WOS:A1982NA53200007

ANTONOV, YA; GRINBERG, VY; ZHURAVSKAYA, NA; SCHMIDT, G; SCHMANDKE, H; TOLSTOGUZOV, VB (1982) MECHANICAL-PROPERTIES AND SOLUBILITY OF FIBER OBTAINED FROM LIQUID 2-PHASE SYSTEMS WATER-CASEIN-SODIUM ALGINATE, NAHRUNG-FOOD, doi: - WOS:A1982ND93500002

NITZSCHE, G; FRITZSCHE, J; ENGLISCH, HG; LIETZAU, H (1981) METHODOLOGY OF BREEDING SYNTHETIC LINES WITH ATTENTION BEING ATTACHED TO MEAT DEPOSITION - DEVELOPED IN BREEDING WORK FOR LINE 150 .1. BREEDING PROGRAM, MULTIPLICATION, AND PRODUCTION EFFICIENCY, ARCHIV FUR TIERZUCHT-ARCHIVES OF ANIMAL BREEDING, doi: - WOS:A1981LQ82700006

KANAVIKAR, CR; LOKANATH, GR; SREENIVASAIAH, PV; RAMAPPA, BS (1981) GENETIC-STUDIES ON SEXUAL DIMORPHISM IN BROILER STRAINS OF CHICKEN, INDIAN JOURNAL OF ANIMAL SCIENCES, doi: - WOS:A1981MW71100010

HSIEH, YPC; PEARSON, AM; MORTON, ID; MAGEE, WT (1980) SOME CHANGES IN THE CONSTITUENTS UPON HEATING A MODEL MEAT FLAVOR SYSTEM, JOURNAL OF THE SCIENCE OF FOOD AND AGRICULTURE, doi:10.1002/jsfa.2740310912 - WOS:A1980KR34900011

GOLOVNJA, RV; ROTHE, M (1980) SULFUR-CONTAINING-COMPOUNDS IN THE VOLATILE CONSTITUENTS OF BOILED MEAT, NAHRUNG-FOOD, doi: - WOS:A1980JM33200004

SCHEIDE, J (1980) CHARACTERISTICS AND FOOD LAW PROBLEMS OF MEAT FLAVORS PRODUCED BY HEAT-TREATMENT, NAHRUNG-FOOD, doi: - WOS:A1980JM33200006

DMITRIENKO, AP; VARFOLOMEEVA, EP; GRINBERG, VJ; TOLSTOGUZOV, VB (1978) MAXIMUM TAKE-UP VELOCITY AS A SPINNABILITY CRITERION AND ITS DEPENDENCE ON COMPOSITION OF COAGULATION BATH, ILLUSTRATED BY AN EXAMPLE OF SODIUM ALGINATE, NAHRUNG-FOOD, doi: - WOS:A1978FE60100003

GRAHAM, JM (1978) CLOSTRIDIUM-BOTULINUM TYPE-C AND ITS TOXIN IN FLY LARVAE, VETERINARY RECORD, doi:10.1136/vr.102.11.242 - WOS:A1978EQ64000009

LEES, P (1977) EXCITING PROSPECTS FOR TRITICALE, JOURNAL OF FLOUR AND ANIMAL FEED MILLING, doi: - WOS:A1977CV94100002

PIRT, SJ (1971) EXPENSIVE MEAT, NATURE, doi:10.1038/231066b0 - WOS:A1971J198300052

REDDY, SG; HENRICKSON, RL; OLSON, HC (1970) INFLUENCE OF LACTIC CULTURES ON GROUND BEEF QUALITY, JOURNAL OF FOOD SCIENCE, doi:10.1111/j.1365-2621.1970.tb01995.x - WOS:A1970I386300020

WATERS, ME (1970) EFFECT OF NITROFURAN-FURYLFURAMIDE-ON CRAB MEAT QUALITY, JOURNAL OF MILK AND FOOD TECHNOLOGY, doi:10.4315/0022-2747-33.8.319 - WOS:A1970H269700005

KINNEY, TB; SHOFFNER, RN (1967) PHENOTYPIC AND GENETIC RESPONSES TO SELECTION IN A MEAT TYPE POULTRY POPULATION, POULTRY SCIENCE, doi:10.3382/ps.0460900 - WOS:A19679776900019
